# Thioholgamide A, a New Anti-Proliferative Anti-Tumor Agent, Modulates Macrophage Polarization and Metabolism

**DOI:** 10.3390/cancers12051288

**Published:** 2020-05-19

**Authors:** Charlotte Dahlem, Wei Xiong Siow, Maria Lopatniuk, William K. F. Tse, Sonja M. Kessler, Susanne H. Kirsch, Jessica Hoppstädter, Angelika M. Vollmar, Rolf Müller, Andriy Luzhetskyy, Karin Bartel, Alexandra K. Kiemer

**Affiliations:** 1 Department of Pharmacy, Pharmaceutical Biology, Saarland University, Campus C2 3, 66123 Saarbrücken, Germany; charlotte.dahlem@uni-saarland.de (C.D.); sonja.kessler@pharmazie.uni-halle.de (S.M.K.); j.hoppstaedter@mx.uni-saarland.de (J.H.); 2 Department of Pharmacy, Pharmaceutical Biology, Ludwig-Maximilians-University of Munich, Butenandtstrasse 5-13, 81377 Munich, Germany; wei-xiong.siow@cup.uni-muenchen.de (W.X.S.); angelika.vollmar@cup.uni-muenchen.de (A.M.V.); karin.bartel@cup.uni-muenchen.de (K.B.); 3 Department of Pharmacy, Pharmaceutical Biotechnology, Saarland University, Campus C2 3, 66123 Saarbrücken, Germany; mariia.lopatniuk@uni-saarland.de (M.L.); a.luzhetskyy@mx.uni-saarland.de (A.L.); 4 Center for Promotion of International Education and Research, Faculty of Agriculture, Kyushu University, 744 Motooka Nishi-ku, Fukuoka 819-0395, Japan; kftse@agr.kyushu-u.ac.jp; 5 Department of Pharmacology for Natural Sciences, Institute of Pharmacy, Martin Luther University Halle-Wittenberg, 06120 Halle, Germany; 6 Department of Microbial Natural Products, Helmholtz Institute for Pharmaceutical Research Saarland (HIPS), Helmholtz Centre for Infection Research, Campus E8 1, 66123 Saarbrücken, Germany; susanne.kirsch-dahmen@helmholtz-hips.de (S.H.K.); rolf.mueller@helmholtz-hzi.de (R.M.); 7 Department of Pharmacy, Saarland University, 66123 Saarbrücken, Germany

**Keywords:** RiPPs, OXPHOS, M2 macrophages, TAM-like macrophages, phagocytosis, thioviridamide-like compounds, scratch assay, mitochondria, migration, qPCR, flow cytometry, HUVEC

## Abstract

Natural products represent powerful tools searching for novel anticancer drugs. Thioholgamide A (thioA) is a ribosomally synthesized and post-translationally modified peptide, which has been identified as a product of *Streptomyces* sp. *MUSC 136T*. In this study, we provide a comprehensive biological profile of thioA, elucidating its effects on different hallmarks of cancer in tumor cells as well as in macrophages as crucial players of the tumor microenvironment. In 2D and 3D in vitro cell culture models thioA showed potent anti-proliferative activities in cancer cells at nanomolar concentrations. Anti-proliferative actions were confirmed in vivo in zebrafish embryos. Cytotoxicity was only induced at several-fold higher concentrations, as assessed by live-cell microscopy and biochemical analyses. ThioA exhibited a potent modulation of cell metabolism by inhibiting oxidative phosphorylation, as determined in a live-cell metabolic assay platform. The metabolic modulation caused a repolarization of in vitro differentiated and polarized tumor-promoting human monocyte-derived macrophages: ThioA-treated macrophages showed an altered morphology and a modulated expression of genes and surface markers. Taken together, the metabolic regulator thioA revealed low activities in non-tumorigenic cells and an interesting anti-cancer profile by orchestrating different hallmarks of cancer, both in tumor cells as well as in macrophages as part of the tumor microenvironment.

## 1. Introduction

Cancer represents the second leading cause of death worldwide, and its incidence and mortality are growing [1]. In search for new antitumor drugs addressing innovative targets, natural products represent powerful tools due to their inherent structures [2]. The recently described natural product thioholgamide A (thioA) belongs to the family of ribosomally synthesized and post-translationally modified peptides (RiPPs), a group of compounds that feature a high structural diversity and that achieve a variety of biological activities [3]. ThioA was identified as a product of *Streptomyces* sp. *MUSC 136T* by genome mining and has been reported to exhibit cytotoxic activities [4].

Increasing evidence indicates that the tumor microenvironment (TME) contributes to the acquisition of hallmarks of cancer traits. These comprise, e.g., sustaining proliferative signaling, evading growth suppressors, resisting cell death, enabling metastasis, reprogramming of energy metabolism, and evading immune destruction [5]. The TME can also substantially influence the efficacy of anticancer therapies [6]. Innate immune cells are highly represented in the TME, with tumor-associated macrophages (TAMs) being the major population [7]. Signals originating from malignant cells and cells of the TME influence the function and phenotype of TAMs. On one end of the multifaceted spectrum of macrophage plasticity, M1 macrophages exhibit a tumor-suppressing response and are usually found in the early phase of tumor formation. During tumor progression, the macrophage population is predominantly skewed towards an M2-like phenotype [8]. This polarization state orchestrates cancer-related inflammation, supports angiogenesis, extracellular matrix remodeling, and tumor cell proliferation. Thereby macrophages promote tumor growth and metastasis [9,10]. A correlation between an increased presence of M2-like TAMs and poor prognosis has been found in various tumor entities, highlighting TAMs as an interesting target in tumor therapy [11].

In this work, the effects of thioA on different hallmarks of cancer were evaluated in 2D and 3D cancer cell in vitro models, in a zebrafish embryo in vivo model, as well as its influence on macrophage phenotypes.

## 2. Results

### 2.1. Thioholgamide A Impairs Tumor Cell Viability and Proliferation

**Figure 1:**
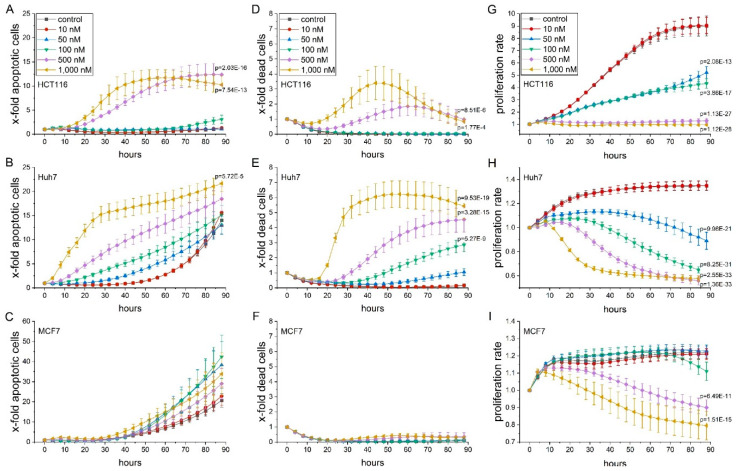
Live cell microscopy-based analysis of thioA-induced cell death and anti-proliferative activity. HCT116, Huh7, and MCF7 cells were stained for caspase 3/7 activity (**A**–**C**) and cell membrane permeability (**D**–**F**) and monitored in an IncuCyte S3 system during thioA or vehicle control treatment over 88 h. Cell confluency was monitored in parallel (**G**–**I**). Fluorescent signals from apoptotic and dead cells were normalized to cell confluency (**A**–**F**). Cell confluency was normalized to time point 0 h (**G**–**I**). Statistical analysis was performed for the last acquired time point using one-way ANOVA followed by Bonferroni’s post-hoc analysis. *n* = 3 (quadruplicates).

The natural product thioA has been shown to reduce tumor cell viability in a set of different tumor cell lines upon a 5-day treatment [4]. We confirmed reduced viability in cancer cell lines from the most abundant and most deadly tumor entities, i.e., breast, liver, colon, and lung [1]. Tumor cell viability was determined after 48 h treatment by MTT assay, leading to IC_50_ values in the nano to low micromolar range (Table 1, Figure S1A). In a 3D-spheroid model, thioA attenuated cell viability as determined by the activity of acid phosphatases (APH, Figure S1B). Since MTT-based assays make use of the metabolic activity as an indirect parameter of cell viability, we further assessed the fractions of cells exhibiting actual markers of cell death in a comprehensive time-dependent live-cell microscopic analysis. We used combined staining for active caspase 3/7 as an indicator of apoptosis and membrane permeability as an indicator of necrosis (Figure 1A–F). Interestingly, in comparison to the low IC_50_ values from MTT measurements, only rather high thioA concentrations and long treatment times provoked the appearance of apoptotic and necrotic markers (for comparison of IC_50_ values see Table 2). Still, apoptosis was induced in concentrations comparable to other apoptosis inducers, such as staurosporine (Figure S2). When comparing IC_50_ values, caspase 3/7 activity- and membrane permeability-based values were several-fold higher than the MTT-based values.

Since thioA induced cell death only at high concentrations, we tested a potential anti-proliferative effect by monitoring cell confluency during treatment using automated microscopy. Notably, the anti-proliferative activity of thioA resulted in the by far lowest IC_50_ values (Figure 1G–I, Table 2). MCF7 cells, devoid of caspase 3 [12], showed a reduced proliferation but no detectable induction of cell death in thioA concentrations up to 1 μM.

**Figure 2:**
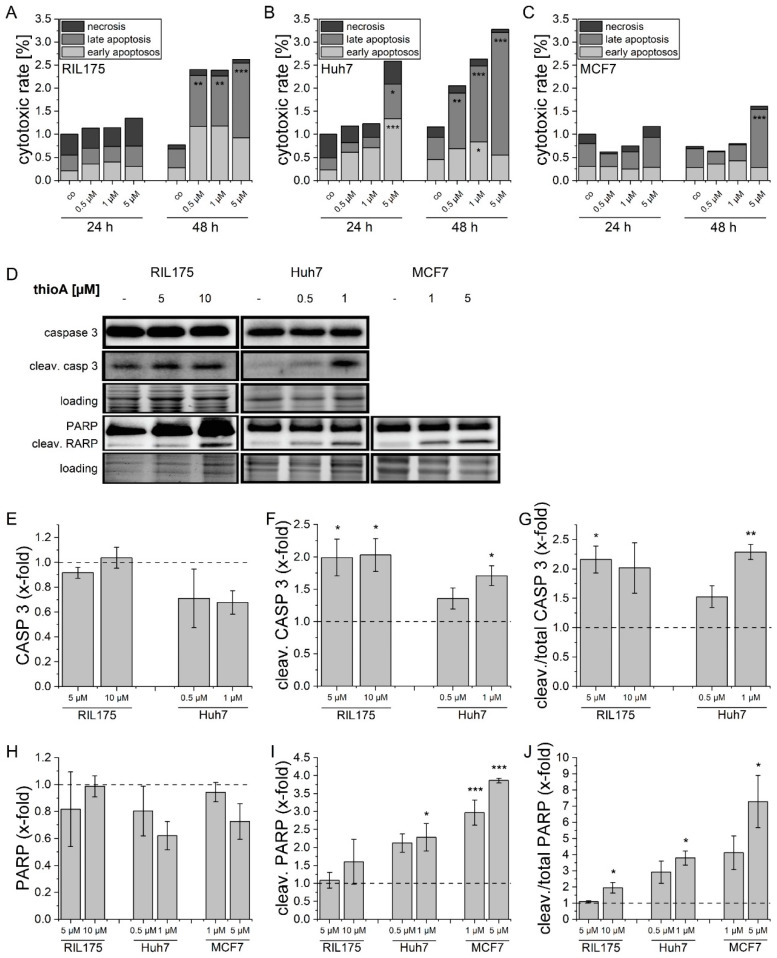
ThioA induces tumor cell death via caspase 3 and PARP cleavage. Proportions of apoptotic, late apoptotic (secondary necrotic), and necrotic cells were assessed in RIL175 (**A**), Huh7 (**B**), and MCF7 (**C**) tumor cells after treatment for 24 h and 48 h by annexin/PI staining and flow cytometry measurements. The gating strategy is shown in Figure S3 Contribution of cleaved caspase 3 and PARP in thioA-induced apoptosis was evaluated by immunoblotting (**D**). ThioA increased levels of cleaved caspase 3 (**E**–**G**) and cleaved PARP (**H**–**J**). Cells were treated for 24 h. One representative Western blot is shown including a total protein loading control. Whole blots are shown in Figure S4. Vehicle control-treated cells were used for data normalization. Statistical analysis was performed using one-way ANOVA and Dunnett post-test; *p* < 0.05 (*), *p* < 0.01 (**), *p* < 0.001 (***). *n* = 3 (triplicates).

In addition to the continuous quantitative assessment of cell death over time, induction of apoptosis was also confirmed via endpoint assays, i.e., flow cytometry and Western blot. These assays confirmed a dose- and time-dependent induction of apoptosis and consecutive secondary necrosis (Figure 2A–C) involving caspase 3 and PARP (Poly(ADP-ribose)-polymerase) cleavage (Figure 2D–J) in concentrations distinctly above IC_50_ values of MTT and proliferation assays. In MCF7 cells, cell death occurred in concentrations about 10-fold higher than IC_50_ value assessed via MTT assays.

Due to the discrepancy between the cytotoxicity expected based on MTT results and that ultimately confirmed by apoptotic and necrotic events as well as the fact that the MTT assay is a metabolic assay, we suggested an influence of thioA treatment on metabolism.

### 2.2. Thioholgamide A Inhibits Oxidative Phosphorylation and Affects Mitochondrial Mass and Morphology

**Figure 3:**
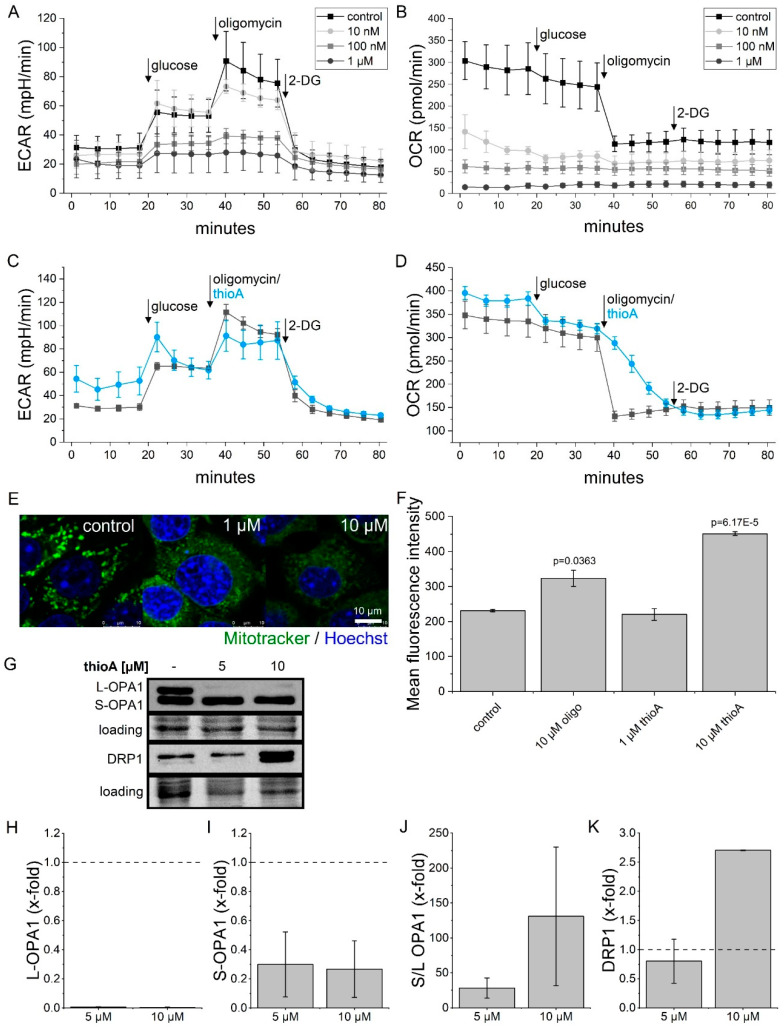
ThioA effects on tumor cell metabolism and mitochondria. Extracellular acidification rate (ECAR) and oxygen consumption rate (OCR) were measured in RIL175 cells in a glycolysis stress test using a Seahorse 96XF instrument. Cells were either pre-treated for 24 h with 10 nM, 100 nM, or 1 μM thioA (**A**,**B**), or the injection of the ATP synthase inhibitor oligomycin (1 μM) was replaced by an injection of 1 μM thioA (**C**,**D**). The mitochondrial morphology of RIL175 cells treated for 24 h with thioA was visualized by Mitotracker Green (mitochondria) and Hoechst (nuclei) co-staining, followed by confocal live cell imaging. Microscopy revealed a diffuse staining (**E**). Mitochondrial mass of RIL175 cells was analyzed via flow cytometry after 24 h treatment with vehicle control, thioA or oligomycin (oligo), respectively (**F**). Expression of the mitochondrial fission markers OPA1 and DRP1 were analyzed by immunoblotting in RIL175 cells after 24 h thioA treatment (**G**). ThioA increased the S/L OPA1 ratio (**H**–**J**) and DRP1 levels (**K**). One representative Western blot is shown including a loading control for total protein. Whole blots are shown in Figure S5. Vehicle control-treated cells were used for data normalization. Statistical analysis was performed using one-way ANOVA and Dunnett post-test. *n* = 3 (triplicates), *n* = 2 for DRP1 quantification.

The Warburg effect represents a well-known metabolic hallmark of cancer cells, i.e., their dependency on glycolysis rather than on oxidative phosphorylation to sustain proliferation, even in the presence of enough oxygen supply. We therefore analyzed the bioenergetic profile of thioA-treated tumor cells using a Seahorse glycolytic stress test. Pretreatment with thioA resulted in reduced responsiveness towards the ATP synthase inhibitor oligomycin (Figure 3A), while the extracellular acidification rate (ECAR) after glucose addition was only affected in high concentrations. Hence, we suggested that there is no major change in glucose uptake capacity but a shutdown of oxidative phosphorylation (OXPHOS) in a dose-dependent manner (Figure 3B). The reductions in basal ECAR and oxygen consumption rate (OCR) occurring at high concentrations are likely to result from secondary effects induced by thioA. The actions on OXPHOS occurred already in concentrations that do not induce cell death and could not be amplified by the ATP synthase inhibitor oligomycin. Since Takase et al. identified the ATP synthase as a target of the RiPP prethioviridamide [13], we hypothesized that thioA shares this mode of action. Therefore, we replaced oligomycin injection by thioA. Indeed, thioA injection resulted in similar profile curves (Figure 3C,D), as seen by an OCR reduction within the first 20 min of treatment (Figure 3D) and a compensatory ECAR increase (Figure 3C). Similar observations had been observed by other small molecules inhibiting ATP synthase [14].

The inhibition of oxygen consumption was comparable to the effect induced by oligomycin, indicating that thioA inhibits mitochondrial function. Analysis of mitochondrial structure by Mitotracker Green staining further demonstrated a morphological change in the mitochondrial network (Figure 3E), indicating a mitochondrial impairment. Along this line, analysis of mitochondrial mass revealed a similar phenotype of thioA and oligomycin. At low concentrations, mitochondrial mass was not significantly changed, yet was increased in higher concentrations. The latter is most likely a compensatory mechanism to account for dysfunctional mitochondria (Figure 3F). Additionally, Western blot analysis of prominent mitochondrial fission regulators OPA1 and DRP1 showed alterations in their cleavage, indicating deregulation in mitochondrial fusion–fission dynamics (Figure 3G–K).

### 2.3. Thioholgamide A Inhibits Tumor Cell Proliferation In Vitro, in Tumor Spheroids, and In Vivo

**Figure 4:**
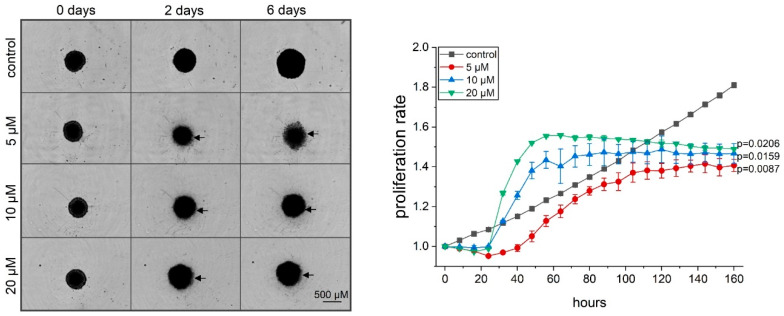
ThioA inhibits proliferation in 3D cell culture. 3-day old HCT116 tumor spheroids were treated with thioA, and the spheroid area was analyzed by automated microscopy (right panel). Treatment with thioA led to a disrupted spheroid structure, as seen by a detachment of cells from the core (arrows, left panel), causing an initial spheroid area increase. Spheroids are shown in representative pictures at the starting point and the time points 2 days and 6 days after treatment (left panel). Statistical analysis was performed for the last acquired time point using one-way ANOVA followed by Bonferroni’s post-hoc analysis. *n* = 2 (quadruplicates).

Our data suggested that attenuated viability in cancer cells upon thioA treatment is due to its actions on tumor cell metabolism (Figure 3) and proliferation (Figure 1), while only to a lower extent on the induction of cell death. To investigate the in vitro anti-proliferative activity more extensively, we made use of a 3D tumor spheroid model. ThioA reduced the growth of spheroids accompanied by a loosened spheroid structure and a detachment of outer cells from the spheroid core (Figure 4), which was not observed for the control treatment with the apoptosis inducer staurosporine (Figure S6). This cell detachment resulted in the detection of an increased area by the automated microscopy at early time points at high concentrations of thioA (Figure 4, right panel).

**Figure 5:**
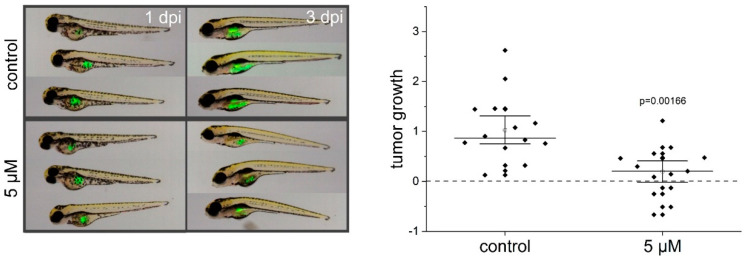
ThioA inhibits proliferation in vivo. Stably enhanced green fluorescent protein (EGFP)-expressing Huh7 cells were injected into the yolk sac of zebrafish embryos 48 hpf (hours post fertilization), followed by treatment with 5 μM thioA or vehicle control in the fish water. Tumor growth was monitored 3 dpi (days post-injection, left panel) via fluorescence imaging, and the tumor area was determined using ImageJ (right panel). Representative pictures are shown. The plot includes individual values and the median line ± 1.5 SEM. Statistical analysis was performed using a two-tailed student’s *t*-test.

As a next step, we used a xenograft zebrafish embryo model to further study the anti-proliferative effects of thioA in vivo. The zebrafish (*Danio rerio*) represents a favorable alternative model for tumor xenograft experiments in accordance with the 3R rules. In addition to immunosuppressed mouse models, it offers advantages, such as the straightforward monitoring of tumor growth in living embryos or the easy application of comparatively small amounts of drugs [15]. In this model, thioA treatment of the tumor cell-injected embryos resulted in significant inhibition of tumor growth (Figure 5). ThioA showed no toxic effects on zebrafish embryos in concentrations up to 10 μM for 72 h, and up to 20 μM for 48 h, as assessed by the observation of eye, heart, and body axis formation, heartbeat, and pigmentation (Figure S7). We further evaluated the toxicity of thioA towards non-tumorigenic human cells using two different in vitro models. Both, primary human umbilical vein endothelial cells (HUVECs) and human serum-differentiated Huh7.5 cells that feature a cell phenotype exhibiting a metabolism similar to normal cells [16], were demonstrated to be less affected by thioA compared to tumor cells (Figure S8). For instance, at 5 μM thioA, which is several-fold higher than the tumor IC_50_ values, over 40% were still alive in primary human endothelial cells.

### 2.4. Thioholgamide A Inhibits Tumor Cell Migration

**Figure 6:**
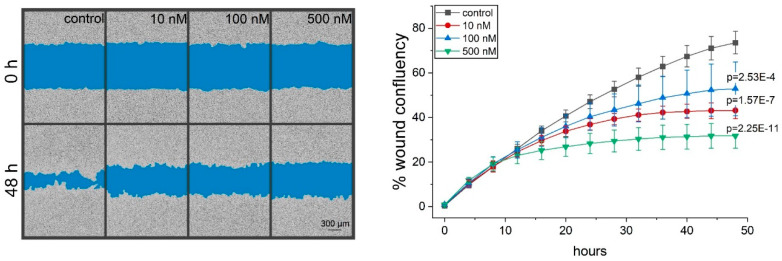
ThioA inhibits cancer cell migration in a scratch wound assay. HCT116 cells were treated with thioA, and wound closure was analyzed in an IncuCyte S3 system over 48 h (right panel). Representative pictures are shown (left panel). Statistical analysis was performed for the last acquired time point using one-way ANOVA followed by Bonferroni’s post-hoc analysis. *n* = 3 (quadruplicates).

In order to test whether thioA affects other hallmarks of cancer, we evaluated the metastatic capacity of tumor cells as modeled by cell migration in a scratch wound assay. Even the very low dose of 10 nM thioA, which showed neither an effect on cell viability nor on proliferation, significantly reduced cell migration of serum-starved tumor cells (Figure 6).

### 2.5. Thioholgamide A Inhibits OXPHOS-Dependent Atp Production in Macrophages

Our data showed effective anti-tumor actions of thioA by affecting tumor cell metabolism. With the importance of macrophages in tumor progression, we analyzed the effects of thioA on the bioenergetic profile of in vitro differentiated and polarized human monocyte-derived macrophages (HMDMs). We investigated five different polarization states of macrophages. Macrophages were generated by either polarizing them in the presence of the M2 cytokines IL4 (M2(IL4)) or IL10 (M2(IL10)) or in the presence of tumor-cell conditioned medium (TAM-like). As comparison, M0 and classically activated M1 macrophages (LPS/IFNγ) were investigated.

**Figure 7:**
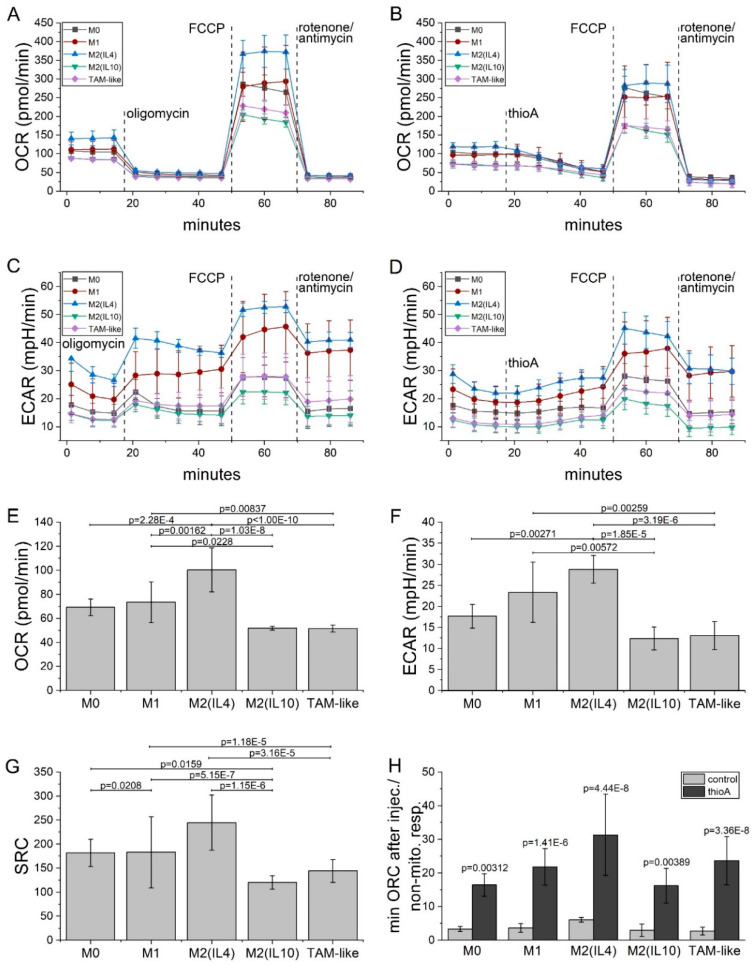
ThioA affects the metabolism of in vitro differentiated and polarized macrophages. Human monocyte-derived macrophages (HMDMs) were polarized into M0, M1, M2(IL4), M2(IL10), and tumor-associated macrophage (TAM)-like macrophages for 24 h. OCR and ECAR were measured in a mito stress test using a Seahorse 96XF instrument. Either 1 μM oligomycin was injected to shut down OXPHOS-dependent ATP production (**A**,**C**), or 1 μM thioA was injected instead (**B**,**D**). Basal OCR (**E**), ECAR (**F**), and spare respiratory capacity (SRC) (**G**) were analyzed for the different macrophage subsets in the oligomycin injected setup. Minimal OCR values at the measurement point 5 (47 min) after respective oligomycin or thioA injection were normalized to the non-mitochondrial respiration (last time point after rotenone/antimycin injection) (**H**). Statistical analysis was performed using one-way ANOVA followed by Tukey’s post-hoc analysis. *n* = 3 (quadruplicates).

On a basal level, M2(IL10) and TAM-like macrophages showed the lowest OCR in Seahorse measurements, while M2(IL4) macrophages had the highest consumption of oxygen (Figure 7A,E). A similar pattern was observed for the subsets in terms of spare respiratory capacity (SRC, Figure 7G), considered as an indicator of how efficiently cells can adapt to changing energy demands [17]. The extracellular acidification rate as an indirect measurement of glycolysis revealed the highest basal levels in M1 and M2(IL4) macrophages (Figure 7C,F). ThioA injection reduced OXPHOS-dependent ATP production (Figure 7B) and caused a compensatory ECAR increase (Figure 7D). In comparison to oligomycin, these effects were achieved more slowly, leading to higher minimal OCR values after injection (Figure 7H). Due to donor-specific differences in the bioenergetic profile of in vitro polarized macrophage subsets, individual graphs are shown in Figure S9.

### 2.6. Thioholgamide A Alters the Macrophage Phenotype

Macrophage phenotypes are linked to their metabolic features. Due to the distinct impact of thioA on macrophage metabolism, we hypothesized an effect on macrophage polarization. In order to determine sub-toxic thioA concentrations for polarized HMDMs, we stained the cells for caspase 3/7 activity and membrane permeability and monitored their viability over 3 days (Figure S10). We chose the concentration of 50 nM for further experiments as it showed no toxic effects in all polarization states during the first 16 h treatment. Potential alterations in the macrophage phenotype were assessed regarding morphology, expression of marker genes, surface markers, as well as phagocytosis.

**Figure 8:**
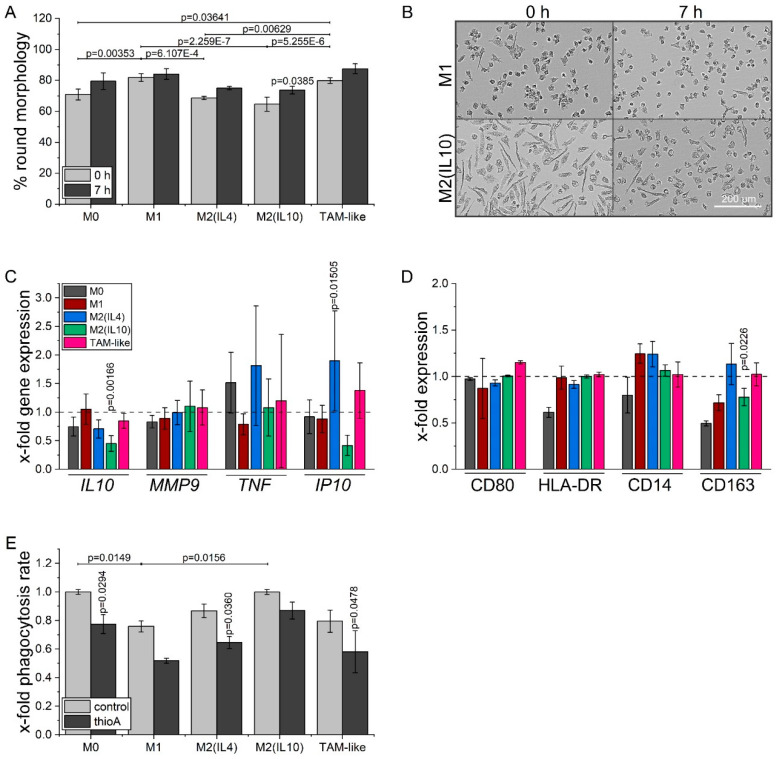
ThioA reduces M2 polarization markers. HMDMs were differentiated and polarized in vitro into M0, M1, M2(IL4), M2(IL10), and TAM-like macrophages for 24 h, followed by 50 nM thioA treatment. Cells were imaged at the beginning and at the end of 7 h treatment using automated microscopy. Cells were grouped based on their eccentricity in an elongated or round phenotype by the IncuCyte cell by cell analysis software (**A**; *n* = 3 (quadruplicates)). Representative pictures of M1 and M2(IL10) macrophages are shown (**B**). Macrophages polarized for 21 h were treated with 50 nM thioA for 7h. Gene expressions of *IL10*, *MMP9*, *TNF*, and *IP10* were measured after treatment by qPCR. Expression values were normalized to *18S* as a housekeeping gene, followed by normalization to vehicle control-treated cells of the respective polarization state (**C**; *n* = 3 (triplicates)). Surface marker expression of M1-associated CD80 and HLA-DR, and M2-associated CD14 and CD163 was analyzed by flow cytometry after a 7 h treatment of macrophages polarized for 21 h. Expression values were normalized to vehicle control-treated cells of the respective polarization state (**D**; *n* = 2 (duplicates)). Macrophages polarized for 24 h were treated with thioA (1 μM) for 30 min, followed by incubation with fluorescent latex beads for 15 min. The proportion of macrophages that engulfed beads was quantified by flow cytometry. Bead-positive macrophages were normalized to M0 cells of the respective donor (**E**; *n* = 3 (duplicates)). Statistical analysis was performed using one-way ANOVA followed by Tukey’s post-hoc analysis.

Macrophage polarization is characterized by distinct morphological features, as seen by a high proportion of elongated cells in M2 macrophages, and predominantly round cells in M1 macrophages. After thioA treatment, M2(IL10) macrophages showed a more M1-like morphology as they comprised a higher proportion of round cells (Figure 8A,B).

Since morphological changes suggested that thioA treatment in sub-toxic concentrations skewed anti-inflammatory macrophages towards a less pronounced M2 phenotype, we analyzed gene expression of polarization markers. We found that thioA caused a reduction in the expression of the anti-inflammatory gene *IL10* in M2-macrophages, while the pro-inflammatory gene *IP10* was induced in M2(IL4) macrophages. The expression of *TNF* and *MMP9* showed no significant alterations (Figure 8C).

Flow cytometry analyses revealed that the M2-associated surface marker CD163 was significantly downregulated in M2(IL10) macrophages by thioA. The expression of the M1 markers CD80 and HLA-DR remained unchanged (Figure 8D).

For the evaluation of macrophage functional activity, the efficiency of phagocytosis was analyzed after thioA treatment by flow cytometry. In this setup, M0 and M2(IL10) macrophages exhibited the highest phagocytic capacity. ThioA decreased phagocytosis in M0, M2(IL4), and TAM-like macrophages (Figure 8E).

## 3. Discussion

The group of RiPPs has attracted increased attention in recent years due to the description of a range of biological activities against bacteria, fungi, and cancer cells. Following the initial observation of apoptosis induction by thioviridamide in 3Y1 cancer cells [18], further anti-cancer actions were described for the thioviridamide-like compounds prethioviridamide [13] and thioalbamide [19] in vitro. We are the first to provide a comprehensive biological profile of the thioviridamide-like RiPP thioA, using 2D- and 3D-cell culture models. To the best of our knowledge, this is the first study describing in vivo anti-tumor activities of a RiPP group member. Most importantly, this study addresses the critical role of thioA in the tumor microenvironment by investigating its effects on the polarization of macrophages as crucial players in the tumor microenvironment.

Using time-resolving automated microscopy approaches, we identified thioA as a potent anti-proliferative agent with IC_50_ values in the low nanomolar range. The anti-proliferative activity was confirmed in an in vivo zebrafish embryo model. The xenotransplantation of human cancer cells into zebrafish embryos extends the scope of early pre-clinical drug testing and offers new opportunities, such as real-time visualization of tumor growth and investigation of new compounds, from which only minor amounts are available [20,21]. In this in vivo model, thioA effectively inhibited tumor growth without showing toxic effects on the embryo. The low toxic activity of thioA was further confirmed in human nontumorigenic cells.

Treatment with concentrations even lower than those inhibiting proliferation resulted in reduced cell migration as another important hallmark of cancer. Another well-known hallmark of cancer is a metabolic reprogramming associated with an increased glycolysis to sustain proliferation even in the presence of enough oxygen supply [22,23]. Even though many cancers preferentially use glycolysis for energy production, the inhibition of OXPHOS, one suggested mechanism of action for thioA, could serve as an attractive anti-cancer target. In fact, different cancer subsets [24] and chemoresistant tumor cells [25] show an increased OXPHOS dependency. Accordingly, different OXPHOS inhibitors are currently under study to investigate their anti-cancer potential in vitro and in vivo [26]. These studies also focus on metabolic plasticity of tumor cells in the context of OXPHOS inhibition. Interestingly, an increased reliance on OXPHOS is not necessarily associated with a dependency on it since cells might still switch towards glycolysis, if necessary. In fact, combination therapy with a glycolytic inhibitor has been shown to sensitize cells towards the OXPHOS inhibitor and thioA derivative prethioviridamide [13].

While thioA acts in nanomolar concentrations on different hallmarks of cancer, distinctly higher concentrations are required to actually induce cell death in cancer cells. Takase et al. identified the ATP synthase as a target of the derivative prethioviridamide, causing the initiation of the integrated stress response (ISR) [13]. The ISR functions primarily as a pro-survival response setting cells into a resting state to handle stress situations, such as amino acid deprivation or ER stress. If homeostasis cannot be restored, apoptotic pathways are finally entered [27].

Our data show that thioA affected mitochondrial morphology and reduced the OCR of treated cells in a dose-dependent fashion to a similar extent as the ATP synthase inhibitor oligomycin. This finding suggests the ATP synthase as a shared target for the derivatives prethioviridamide [13] and thioA. The subsequent ISR initiation would explain the mainly anti-proliferative action of thioA, as cells first enter a resting state before they undergo apoptosis. Interestingly, Takase et al. (2019) did not investigate whether prethioviridamide in fact induced cell death but reported effects on cell viability in a metabolic assay similar to the MTT assay we employed.

In addition to the challenges in the development of potent anti-cancer therapies driven by the complexity of malignant cells themselves, the TME actively contributes to their efficiency. The TME is composed of tissue-resident and a large proportion of recruited immune cells, with macrophages playing a central regulatory role [28]. The beneficial effect of anti-cancer agents targeting both tumor cells and macrophages has been shown for the natural compound trabectedin. The compound originally isolated from *Ecteinascidia turbinata* was initially approved as an anti-proliferative drug for the treatment of advanced soft tissue sarcoma [29]. The drug was later demonstrated to reduce the number of tumor-infiltrating macrophages [30]. This supplementary property substantially contributes to the activity of this clinically useful anti-cancer agent.

Besides TAM depletion and inhibition of recruitment, a reprogramming of TAMs represents a promising approach [6]. Shifting TAM polarization from a tumor-supporting (M2) towards an actively tumor-rejecting (M1) phenotype would rebalance the TME and effectively support anti-tumor strategies. In this context, we focused the biological profiling of thioA not only on tumor cells but also investigated its effect on the polarization, viability, and activity of different macrophage subsets. The paradigm of a rather clear cut between M2 macrophages supporting cancer and M1 macrophages antagonizing cancer has been challenged by recent sequencing studies of immune cells from the TME, and TAMs exhibited some characteristics of both M1 and M2 polarization depending on localization and tumor stage [31,32]. In fact, TAM-like macrophages displayed an intermediate phenotype regarding, e.g., morphology and phagocytosis in our study.

As demonstrated in tumor cells, thioA inhibited OXPHOS-dependent ATP production also in macrophages. The link between macrophage metabolism and their phenotypes has become a highly studied focus of research in recent years. It has been shown that inflammatory M1 macrophages have an enhanced glycolytic metabolism to fulfill their energy demands, while anti-inflammatory macrophages mainly rely on OXPHOS [33,34]. Our bioenergetic data of in vitro polarized HMDMs revealed an overall quiescent profile for M0, M2(IL10), and TAM-like macrophages, as indicated by low basal OCR and ECAR values. M1 as well as M2(IL4) macrophages showed higher metabolic activities, even though macrophages from different donors responded to varying degrees to the respective polarization stimuli. The slower OCR reduction caused by thioA compared to oligomycin might be caused by a lower cell penetration or target binding affinity.

Macrophage subtypes also differed in their SRC, which is defined as the difference between the basal and the maximal respiration. As many cells operate at a basal level that only requires a part of their total metabolic capacity, the SRC provides information on how the cell can deal with changing energy demands and withstand periods of stress [17]. The low SRC of M2(IL10) and TAM-like macrophages implies an increased sensitivity of these cells towards an OXPHOS inhibition. The fact that these cells also show just a small degree of subsequent glycolysis increase after thioA or oligomycin injection leads to the conclusion that M2(IL10) and TAM-like macrophages might have a specific reaction in response to the inhibition of OXPHOS.

To test the implications of thioA-mediated modulation of macrophage metabolism on polarization state, we used a thioA concentration that mainly inhibited tumor cell proliferation without showing distinct cytotoxic activities against either tumor cells or macrophages during the observed treatment periods. Overall, this low concentration of 50 nM, thioA weakened the extent of M2 polarization. ThioA reduced the anti-inflammatory polarization marker *IL10* [35,36] on gene expression levels in M2 macrophages and increased the pro-inflammatory *IP10* [37] in M2(IL4) and TAM-like macrophages. The expression of the M2-associated surface marker CD163 [38,39] was also reduced after treatment in M2(IL10) macrophages. Moreover, morphology was skewed towards an M1-like phenotype during treatment, which has also been described after oligomycin treatment of mouse macrophages [40].

Tumors are often referred to as “wounds that never heal”. In this context, apoptotic cells inside the tumor can polarize macrophages into an M2 status, which in turn try to maintain tissue homeostasis by engulfing cell debris, promoting tissue repair, and resolving inflammatory reactions [41,42,43]. Hence, this phenotype comprises a high efficiency in the phagocytosis of apoptotic cells [44,45,46], while inflammatory and microbicidal M1 macrophages excrete a preferential engulfment of bacteria [47,48]. In this study, M2 macrophages exhibited a higher phagocytic activity compared to M1 macrophages, which was reduced after thioA treatment in all phenotypes. The inhibition of phagocytosis after OXPHOS inhibition was described before for neutrophils [49].

## 4. Materials and Methods

### 4.1. Cell Culture

Tumor cell lines were cultured in RMPI-1640 (Huh7, A549) or DMEM (HCT116, MCF7, Ril175) medium, supplemented with 10% FCS, 100 U/mL penicillin/streptomycin, and 2 mM glutamine. Media and supplements were purchased from Sigma-Aldrich (St. Louis, MO, USA) (#R0883, #D6546, #F7524, #P433, #G7513). The cells were maintained at 37 °C in a humidified atmosphere of 5% CO_2_.

For spheroid formation, 3000 HCT116 cells were seeded into low attachment U-bottom plates (Band #781900). Three-day old spheroids were used for further experiments.

To generate stably EGFP-expressing Huh7 cells, cells were transfected with the empty backbone mEGFP-C1 plasmid (Addgene, Watertown, MA, USA, plasmid #54759) using lipofectamine 3000 (Thermo Fisher Scientific, Waltham, MA, USA, #L3000008) according to the manufacturer’s protocol. Forty-eight hours after transfection, medium was changed, and the selection antibiotic geneticin (Thermo Fisher Scientific #11811023) was added in a concentration of 1 mg/mL for further cultivation. Cells were used for xenograft injections after at least 3 sub-culturing steps, and stable EGFP expression was controlled by flow cytometry. Culturing without geneticin for 3 days did not change EGFP expression.

For human serum differentiation, 3000 Huh7.5 cells were seeded per well into 96-well plates in RPMI-1640 full growth medium. The next day, medium was changed for RPMI-1640 supplemented with 2% human serum (PAN biotech, Aidenbach, Germany, #P40-2701) instead of FCS. Medium was changed twice a week, for 3 weeks. Differentiated cells displayed alterations in morphology from a spindle-shaped to a cobble-stone phenotype, associated with a switch from unrestrained growth to contact inhibition, which was described to be linked to an induction of hepatocyte-specific genes [16].

Primary human umbilical vein endothelial cells (HUVECs) were isolated and cultured as described previously [50].

### 4.2. Cultivation of Human Monocyte-Derived Macrophages

Buffy coats were obtained from healthy donors (Blood Donation Center, Saarbruecken, Germany), authorized by the local ethics committee (State Medical Board of Registration, Saarland, Germany; permission no. 173/18). Peripheral blood mononuclear cells (PBMC) were isolated by density gradient centrifugation using Lymphocyte Separation Medium 1077 (PromoCell, Heidelberg, Germany, #C-44010) in Leucosep tubes (Greiner, Kremsmünster, Austria, #227290). PBMCs were sorted for CD14 positive cells by positive selection using CD14 magnetic beads (Miltenyi, Bergisch Gladbach, Germany, #130-050-201). Sorted monocytes were seeded at a density of 0.5 × 10^6^ cells/mL, and differentiated in full growth RPMI-1640 media supplemented with 20 ng/mL human recombinant macrophage colony-stimulating factor (M-CSF, Miltenyi #130-096-492) for 6 days. To polarize the human monocyte-derived macrophages (HMDMs) in vitro, the differentiation media was supplemented with 20 ng/mL recombinant IFNγ (Miltenyi #130-096-484) and 100 ng/mL LPS (Ultrapure LPS from Escherichia coli K12 #tlrl-peklps) for M1 polarization; either 20 ng/mL IL4 (Miltenyi #130-093-921) or IL10 (Miltenyi #130-093-948) for M2 polarization; or left without further supplementation for M0 macrophages. TAM-like macrophages were generated by cultivation in tumor conditioned media (TCM) supplemented with 20 ng/mL M-CSF. For TCM production, 20 mL of media was incubated with a confluent cell layer of A549 cells in a T75 cell culture flask for 48 h, following sterile filtration to remove cell debris. In all experiments comparing macrophage subsets, cells were differentiated and polarized from monocytes obtained from the same donor.

### 4.3. Thioholgamide A Isolation and Purification

The thioA biosynthetic gene cluster within the integrated plasmid pTho was expressed in *S. lividans* ΔYA8 [51] heterologous host strain. The strain *S. lividans* ΔYA8: pTho was inoculated in 15 mL of the TSB (Tryptic soy broth 30 g/L) vegetative media and cultivated for 2 days at 28 °C, 180 rpm. Then, 2 mL of the vegetative culture was transferred into a 300 mL baffled flask with 50 mL of DNPM production media (Soytone 7.5 g/L, baker’s yeast 5 g/L, MOPS 21 g/L, dextrin 40 g/L, pH 6.8) and cultivated for 6 days at 28 °C, 180 rpm. The extraction was performed with an equal amount of n-butanol. The butanol extract was then evaporated, and the dry residue was dissolved in methanol and used for subsequent purification.

The obtained methanol extract was used for purification of the compound on a 1-m-long column packed with cross-linked dextran polymer beads. Methanol was used as a solvent for elution. Fractions were collected every 14 min with a flow rate of 1 mL/min. Each portion was analyzed for the presence of thioA on HPLC coupled with an MS part (amaZon SL) using a C18 column (linear gradient from 5% to 95% acetonitrile). The fractions containing thioA were pooled, evaporated, and dissolved in methanol. Afterward, the thioA enriched methanol extract was further purified on a Dionex Ultimate 3000 preparative HPLC using a C18 column (linear gradient from 5% to 95% acetonitrile). The fractions with pure thioA were again evaporated and further used for bioassays.

### 4.4. Endotoxin Assay

Prior to cell treatments, thioA was tested for the absence of endotoxins using the Endozyme II assay kit (Biomérieux, Marcy-l’Étoile, France, #890030) according to manufacturer’s instructions.

### 4.5. Viability Measurements

#### 4.5.1. MTT Assay

For viability assays based on MTT reduction, cells were seeded in appropriate numbers to reach confluency the next day. They were treated with thioA in different concentrations for 48 h. The stock solution of thioA was prepared in DMSO, and solvent controls were tested concurrently. The viability of adherent cells was determined by replacing the supernatants with 0.5 mg/mL MTT (3-(4,5-dimethylthiazole-2-yl)-2,5 diphenyltetrazolium bromide, Sigma-Aldrich #M5655) solution in respective culture media. After 30 min incubation, cells were lyzed in DMSO, and the absorbance was measured at 560 nm using a microplate reader (GloMax™). IC_50_ values were calculated by non-linear regression using OriginPro^®^.

#### 4.5.2. APH Assay

The viability of HCT116 cells forming tumor spheroids was analyzed in an acid phosphatase assay, measuring phosphatase activity. Spheroids (3 days old) were treated for 48 h with thioA or vehicle control before the supernatant was replaced by 100 μL assay buffer (0.1 M sodium acetate (pH 5.2), 0.1% (V/V) Triton X-100 in H_2_O, supplemented freshly with 4 mg/mL p-nitrophenyl phosphate (final pH 4.8, Thermo Fisher Scientific #34045). Spheroids were incubated for 1.5 h at 37 °C before 10 μl 1 M NaOH was added, and absorption was measured at 405 nm on a microplate reader (GloMax^™^).

#### 4.5.3. Annexin V/PI Staining

The flow cytometry-based analysis of apoptotic and necrotic cell death was performed with cells harvested after the indicated treatment period and subsequent staining with eBioscience^™^ Annexin V-FITC Apoptosis Detection Kit (Invitrogen, Carlsbad, CA, USA, #BMS500FI-100) according to the manufacturer’s protocol. Stained cells were analyzed by flow cytometry (Canto II, Beckton Dickinson (BD), Franklin Lakes, NJ, USA). Cells positive for Annexin V staining were considered as early apoptotic, PI-positive cells as necrotic, and both positive as late apoptotic (Figure S3).

#### 4.5.4. Time-Dependent Cell Death Measurement

For the analysis of time-dependent effects, cells were analyzed in an IncuCyte S3 System (Sartorius, Göttingen, Germany). Supernatant was replaced by the respective media containing IncuCyte Cytotox Red (Sartorius #4632) and Caspase-3/7 Green (Sartorius #4440) reagents according to the manufacturer’s instructions. Cells were treated with different concentrations of thioA, staurosporine or DMSO vehicle control, and cell confluency as well as apoptotic and necrotic events were monitored for 3 days. Apoptotic and cytotoxic IC_50_ values were calculated based on Caspase-3/7 Green or Cytotox Red positive cells by non-linear regression using OriginPro^®^.

### 4.6. Western Blot

Cells were harvested and lysed in RIPA lysis buffer containing a protease inhibitor mix (Roche, Basel, Switzerland, #4693159001). Lysates were centrifuged at 10,000× *g* for 10 min and 4 °C. Protein amounts were assessed by Bradford assay, and an equal amount of protein was separated by SDS-PAGE and transferred to nitrocellulose membranes (Hybond-ECLTM, Amersham Bioscience, Amersham, UK). BSA (5%) in PBS with 0.1% Tween 20 was used as a blocking buffer for 1 h, and membranes were incubated with anti-PARP (1:1000, Cell Signaling, Cambridge, UK, #9542), anti-caspase 3 (1:1000, Santa Cruz, Dallas, TX, USA, #sc-7148), or anti-active caspase 3 (1:1000, Sigma Aldrich, St. Louis, MO, USA, #C8487), OPA1 (1:1000, Cell Signaling #80471) or DRP1 (1:1000, Cell Signaling #8570) primary antibodies at 4 °C overnight. Secondary antibodies were incubated accordingly and subsequently conjugated with horseradish peroxidase and freshly prepared ECL solution, which contained 2.5 mM luminol. Conjugated proteins were detected by the ChemiDoc^™^ Touch Imaging System (Bio-Rad, Hercules, CA, USA) and quantified by ImageLab software. For quantification, protein amount was normalized to total protein loading and detected by 2,2,2-trichloroethanol activation as described previously [52,53].

### 4.7. Mitochondrial Mass

Cells were treated as indicated for 24 h and incubated with Mito-Tracker^TM^ Green FM (100 nM, Thermo Fisher Scientific #M7514) for 30 min. Cells were harvested and mean fluorescence intensity of Mito-Tracker^TM^ staining was analyzed by flow cytometry (Canto II, Beckton Dickinson). The number of 30,000 events was collected for each sample.

### 4.8. Seahorse Measurement

The cellular glycolysis stress and mito stress tests were performed using an Agilent Seahorse 96XF device and respective kits. The assays were performed as described in the manufacturer’s protocol (#103020-400, #103015-100). In brief, the cells were seeded and pre-treated with thioA if indicated. The medium was replaced by seahorse medium one hour prior to measuring. For the glycolysis stress test, 20,000 RIl175 cells were seeded and treated with 10 mM glucose, 1 μM oligomycin, and 50 mM 2-desoxyglucose. Macrophages were analyzed using the mito stress test. Macrophages were differentiated for 6 days, and 120,000 cells were seeded per well. Macrophages were polarized for 24 h prior to measurements and treated with either 1 μM oligomycin or 1 μM thioA, 2 μM FCPP, and 0.5 μM Rotenon/Antimycin A. The data were analyzed by the Seahorse Wave Software (Agilent Technologies, Santa Clara, CA, USA).

After Seahorse measurements, cells were stained with Hoechst dye and fluorescence intensity was analyzed in a plate reader to ensure an equal cell distribution also after different treatment steps. Since no significant changes were observed (Figure S11), ORC and ECAR values were analyzed without further normalization.

### 4.9. Immunofluorescence

Cells were seeded on μ-Slide 8 Well for 24 h and treated as indicated. The cells were rinsed with PBS and incubated with MitoTracker^™^ Green FM (100 nM, Thermo Fisher Scientific #M7514) for 30 min. The nuclei were stained by Hoechst 33342 (2.5 μg/mL) for 30 min. The slides were mounted with mounting buffer prior to the insertion of the coverslip. All images were observed by fluorescence microscopy (Leica SP8 Inverted Scanning Confocal Microscope).

### 4.10. Rna Isolation, Reverse Transcription, and Quantitative Pcr

In vitro differentiated and polarized HMDMs (polarized for 21 h) were treated for 7 h with 50 nM thioA or solvent control, respectively. Total RNA from treated cells was isolated using the High Pure RNA Isolation Kit (Roche #11828665001). Equal amounts of RNA were transcribed with the High Capacity cDNA Reverse Transcription Kit (Thermo Fisher Scientific #4368813) in the presence of an RNase inhibitor (Invitrogen #10777-019) according to manufacturer’s instructions. cDNA was analyzed in qPCR using 5xHotFirePol EvaGreen qPCR Mix (Solis BioDyne, Tartu, Estonia, #08-24-00020) and the primers listed in Table 3. The PCR was performed in a CFX96 touch^™^ Real-Time PCR detection system (Bio-Rad). Data were normalized to the housekeeping gene *RNA18S*.

### 4.11. In Vitro Proliferation Measurement

For the kinetic proliferation analysis, HCT116 and HuH7 cells were treated with different concentrations of thioA, and their proliferation was observed in an IncuCyte S3 System. Proliferation inhibitory IC_50_ values were calculated by non-linear regression using OriginPro^®^. The growth of HCT116 spheroids was analyzed using the IncuCyte spheroid analyzer. Spheroids (3 days old) were treated with thioA, and the spheroid area was determined over 6 days.

### 4.12. Zebrafish Xenograft Model And In Vivo Proliferation Measurement

AB wild-type zebrafish embryos were used for tumor cell injections. Zebrafish husbandry and all experiments were performed in accordance with the European Union Directive on the protection of animals used for scientific purpose (Directive 2010/63/EU) and the German Animal Welfare Act (§11 Abs. 1 TierSchG) and maintained using standard methods [54]. Adult zebrafish were kept in the automated aquatic eco-system (PENTAIR, Apopka, UK) and monitored regularly: temperature (27 ± 0.5 °C); pH (7.0 ± 0.1); conductivity (800 ± 50 μS); light-dark cycle (14 h–10 h). Fish were fed twice a day with dry small granulate food and freshly hatched live *Artemia Cysts* once a day.

For experiments, embryos were kept at 28 °C in 0.3× Danieau’s solution (17 mM NaCl, 2 mM KCl, 1.5 mM HEPES, 1.8 mM Ca(NO_3_)_2_, 0.12 mM MgSO_4_). At 48 hpf, embryos were dechorionized manually, anesthetized using 168 μg/mL tricaine (Sigma-Aldrich #A5040), and 1 nL tumor cell suspension was injected into the yolk sac using a FemtoJet microinjector (Eppendorf, Hamburg, Germany). Stably EGFP-expressing Huh7 cells were suspended in PBS containing 0.1% BSA, and 2000 cells were injected per embryo. Single embryos were placed into 96-well plates and incubated with 0.3× Danieau’s solution containing solvent control or 5 μM thioA. On the next day, embryos were sorted for tumor formation and monitored with a Leica M205 FCA fluorescence stereomicroscope. Tumor growth was determined by imaging 3 days post-injection (dpi). The growth rate was calculated as follows: (tumor area 3 dpi − tumor area 1 dpi)/tumor area 1 dpi.

The effects of thioA on zebrafish embryo development and viability was assessed in 24 hpf embryos. Embryos were treated with thioA or vehicle control in the fish water. Eye, heart, and body axis formation, heartbeat, and pigmentation were observed microscopically during 72 h treatment. Embryos were euthanized not later than 5 days post fertilization.

### 4.13. Migration Measurement

To analyze the migration of HCT116 cells in the IncuCyte system, 80,000 cells per well were seeded into an ImageLock 96-well plate. The next day, scratches were conducted using the Woundmaker tool (IncuCyte Migration Kit). Cells were washed twice with 2% FCS containing media, which was also used for further cultivation. Cells were treated with thioA, and the migration was monitored for 48 h. The cell covered wound area was analyzed and quantified using the IncuCyte migration software.

### 4.14. Macrophage Surface Marker Expression

HMDMs were isolated, differentiated, and polarized as described above for 24 h. Polarized cells were treated with 50 nM thioA for 7 h. Cells were detached using accutase solution (Sigma-Aldrich #A6964), washed, and resuspended in FACS wash (PBS, 2.5% FCS, 0.1% sodium azide). Cells were blocked in human Fc Block (BD, #564220) for 15 min, and stained for 30 min on ice with anti-CD14-APC (BD #555399), anti-CD163-PE-CF594 (BD #562670), anti-CD80-BB515 (BD #565008), and anti-HLA-DR-PerCP-CY5.5 (BD #560652) antibodies. After washing, stained cells were resuspended in 1% paraformaldehyde in PBS prior to flow cytometric analysis on a BD LSRFortessa. Data were analyzed using BD FACSDiva software (BD Biosciences, San Jose, CA, USA). Median fluorescence intensity of singlet cells was used to quantify surface marker expression.

### 4.15. Macrophage Morphology Analysis

HMDMs were isolated, differentiated, and polarized as described above for 24 h. Polarized cells were treated with 50 nM thioA for 7 h. In parallel, cells were imaged at the beginning and the end of treatment in an IncuCyte system. Afterward, cells were analyzed for their morphology with the IncuCyte analysis software and grouped in a round and elongated phenotype based on their eccentricity.

### 4.16. Macrophage Phagocytosis Assay

HMDMs were isolated, differentiated, and polarized as described above for 24 h. Polarized cells were treated with 1 μM thioA for 30 min. After incubation for 15 min with fluorescent latex beads (50 beads/cell, Fluoresbrite carboxylated YG microspheres, 1.75 μm, Polyscience, Warrington, England, UK, #17687), macrophages were washed four times with cold PBS and detached from plates using PBS containing 5 mM EDTA. Cells were resuspended in FACS wash and examined on a BD LSRFortessa using BD FACSDiva software (BD Biosciences).

### 4.17. Statistical Analysis

The data are expressed as mean ± SEM (standard error of the mean) of at least 3 independent experiments performed in replicates, if not indicated otherwise. Statistical differences between two groups were calculated using a two-tailed Student’s *t*-test; one-way Analysis of Variance (ANOVA) analysis followed by Tukey’s, Bonferroni’s, or the Dunnett post-hoc analyses was used for statistical comparison of more than two groups. Calculations were performed using the OriginPro^®^ 2020 software.

## 5. Conclusions

In conclusion, thioA exhibits an interesting biological profile for new tumor therapeutic strategies. As a metabolic regulator, it can play a pivotal role in orchestrating different hallmarks of cancer in cancer cells and macrophages. In combination with its low toxicity in non-tumorigenic cells and in vivo, thioA represents an interesting candidate for further preclinical testing.

## Figures and Tables

**Table 1:** IC_50_ values of thioholgamide A (thioA) against a panel of tumor cell lines measured in the metabolic viability MTT assay after 48 h treatment.

Cell Line	IC_50_
HCT116	176 nM
Huh7	141 nM
MCF7	480 nM
A549	1.16 μM
RIL175	157 nM

**Table 2:** IC_50_ values (nM) ± SEM of thioA against HCT116, Huh7, and MCF7 tumor cell lines based on caspase 3/7 activity, membrane permeability, and cell confluency measured by automated microscopy; calculated for 48 h treatment.

Cell Line	Caspase 3/7 Activity	Membrane Permeability	Proliferation
HCT116	412.7 ± 27.5	840.8 ± 163.6	90.8 ± 5.5
Huh7	197.2 ± 44.8	578.8 ± 55.8	52.1 ± 5.7
MCF7	n.d.	n.d.	489.9 ± 9.3

n.d. (not detectable in concentrations up to 1 μM thioA).

**Table 3:** Primer sequences used for qPCR.

Gene	Accession Number	Primer Forward Sequence	Primer Forward Sequence
*IL10*	NM_000572	CAACAGAAGCTTCCATTCCA	AGCAGTTAGGAAGCCCCAAG
*MMP9*	NM_004994.3	CTTTGAGTCCGGTGGACGAT	TCGCCAGTACTTCCCATCCT
*TNF*	NM_000594.4	CTCCACCCATGTGCTCCTCA	CTCTGGCAGGGGCTCTTGAT
*IP10*	NM_001565.4	GAGCCTACAGCAGAGGAACC	AAGGCAGCAAATCAGAATCG
*RNA18S*	NR_003286.4	AGGTCTGTGATGCCCTTAGA	GAATGGGGTTCAACGGGTTA

## Supplementary Materials

The following are available online at https://www.mdpi.com/2072-6694/12/5/1288/s1, Figure S1: ThioA-induced effects on tumor cell viability, Figure S2: Live cell microscopy-based analysis of staurosporine (STU)-induced cell death, Figure S3: Gating strategy for flow cytometric analysis of apoptotic (EA), late apoptotic (LA), and necrotic (NC) tumor cells after thioA or vehicle control treatment for 48 h assessed by annexin/PI staining summarized in Figure 2A–C, Figure S4: Whole blots and corresponding stain-free gels with densitometric readings summarized in Figure 2D–F, Figure S5: Whole blots and corresponding stain-free gels with densitometric readings summarized in Figure 3G–K, Figure S6: Staurosporine-induced effects on proliferation in 3D cell culture, Figure S7: ThioA effects on zebrafish embryo development and viability, Figure S8: ThioA-induced effects on normal cell viability, Figure S9: ThioA affects the metabolism of in vitro differentiated and polarized macrophages; donor specific differences. HMDMs were polarized into M0, M1, M2(IL4), M2(IL10), and TAM-like macrophages for 24 h, Figure S10: Assessment of toxic concentrations of thioA in HMDMs, Figure S11: Cell quantification after Seahorse experiments by Hoechst staining.
