# Supplementary material for: Thioholgamide A, a New Anti-Proliferative Anti-Tumor Agent, Modulates Macrophage Polarization and Metabolism
Source: Cancers (Basel). 2020 May 19;12(5):1288. doi: 10.3390/cancers12051288 (PMC7281193; doi:10.3390/cancers12051288)
Supplement: Supplementary file 1 [file cancers-12-01288-s001.pdf]

Supplementary material

Thioholgamide A, a New Anti-Proliferative Anti-Tumor Agent, Modulates Macrophage Polarization and Metabolism

Charlotte Dahlem, Wei Xiong Siow, Maria Lopatniuk, William Ka Fai Tse, Sonja M Kessler, Susanne H Kirsch, Jessica Hoppstädter, Angelika M Vollmar, Rolf Müller, Andriy Luzhetskyy, Karin Bartel and Alexandra K Kiemer

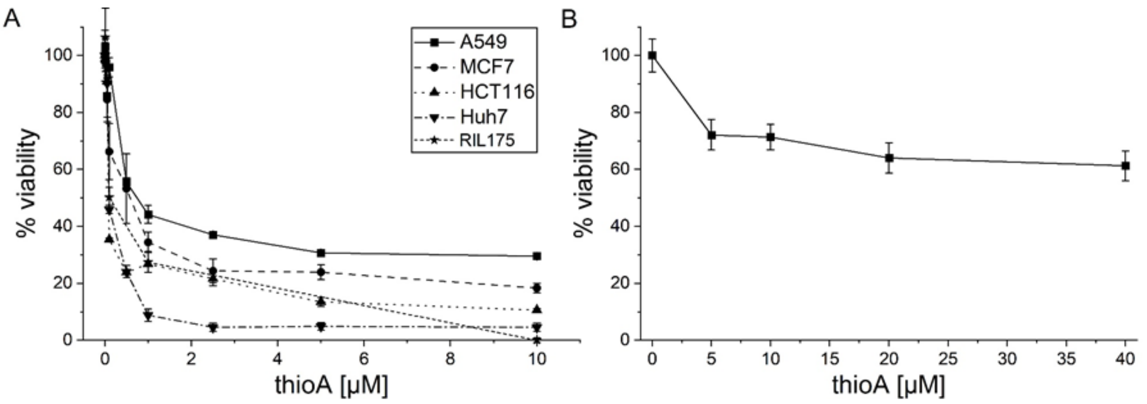

Figure S1. ThioA-induced effects on tumor cell viability. A set of tumor cells was treated with increasing concentrations of the compound, and cell viability was determined after 48 h treatment in an MTT assay (**A**). 3-day old HCT116 spheroids were treated with thioA for 48 h, followed by an APH assay to assess viability, (**B**). Vehicle control-treated cells were used for data normalization.

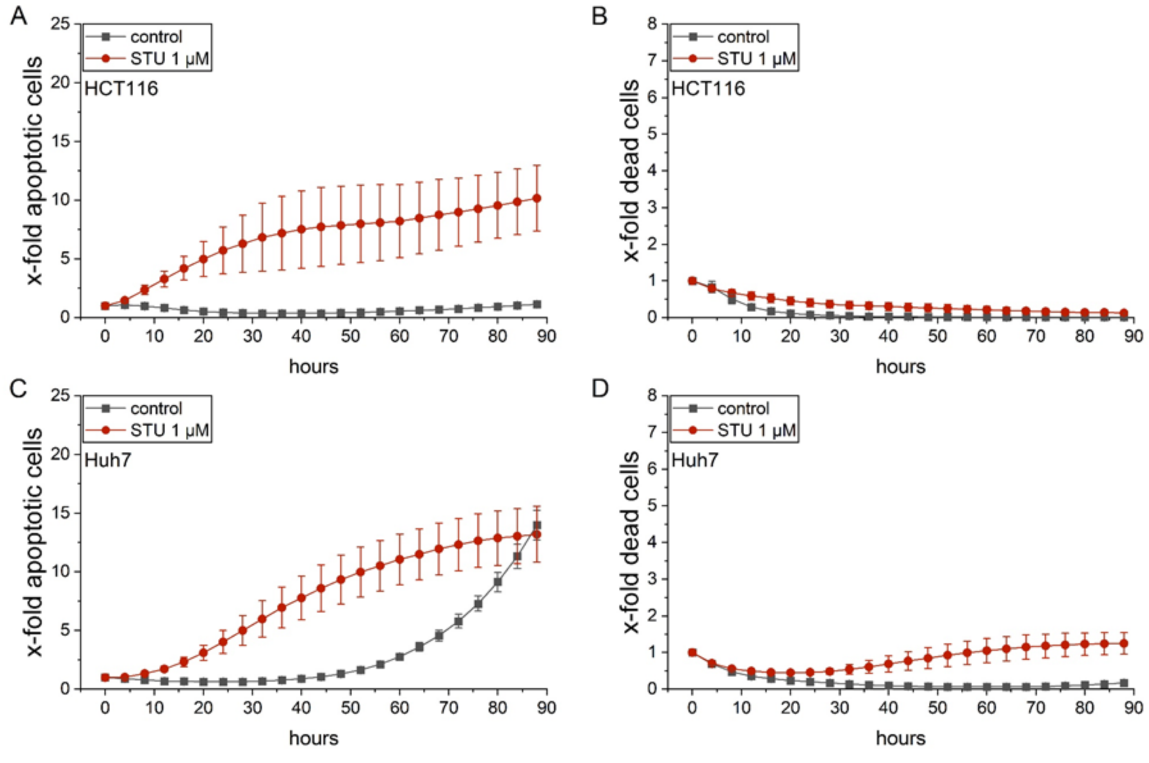

Figure S2. Live cell microscopy-based analysis of staurosporine (STU)-induced cell death. HCT116 and Huh7 cells were stained for caspase 3/7 activity (apoptotic cells, **A**, **C**) and cell membrane

permeability (dead cells, **B**, **D**), and monitored in an IncuCyte S3 system during STU or vehicle control treatment over 88 h. Fluorescent signals from apoptotic and dead cells were normalized to cell confluency. n = 3 (triplicates).

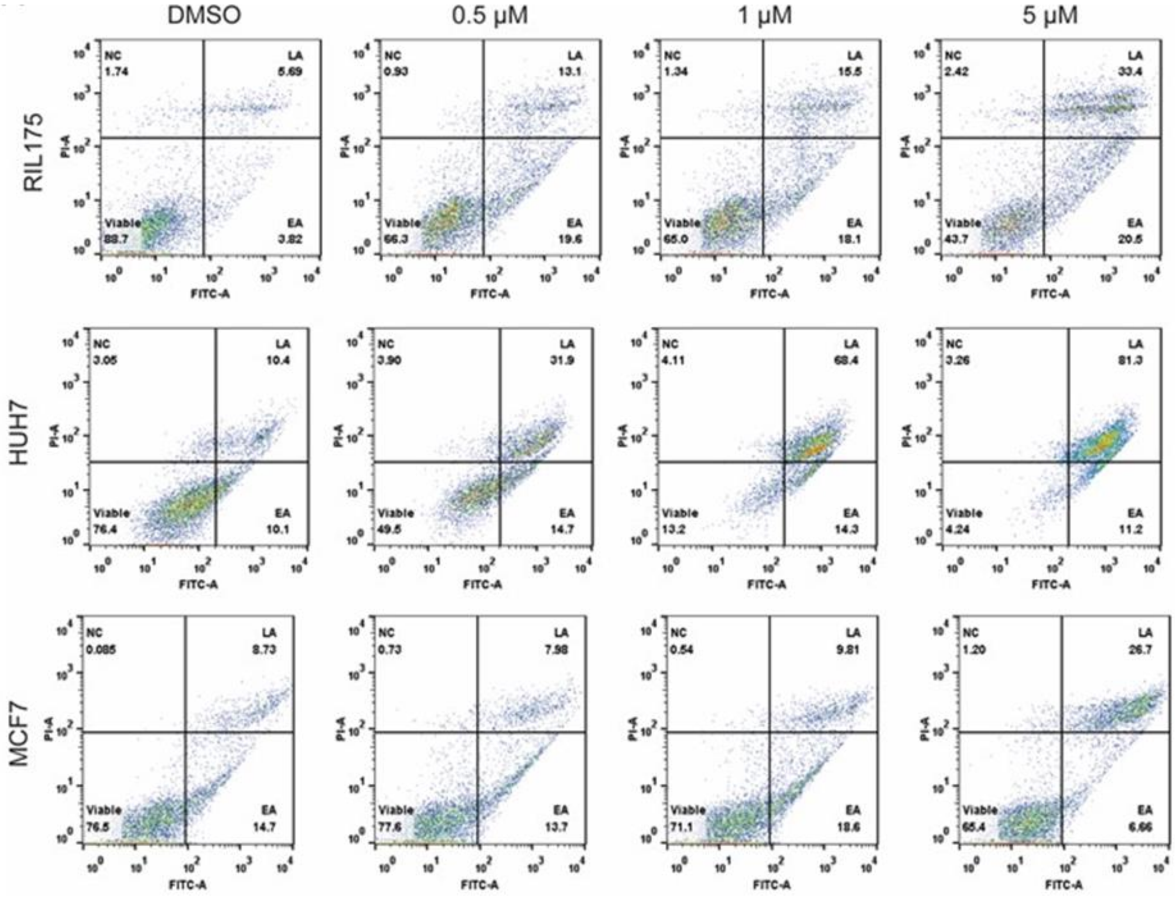

Figure S3. Gating strategy for flow cytometric analysis of apoptotic (EA), late apoptotic (LA), and necrotic (NC) tumor cells after thioA or vehicle control treatment for 48 h assessed by annexin/PI staining summarized in Figure 2 A–C.

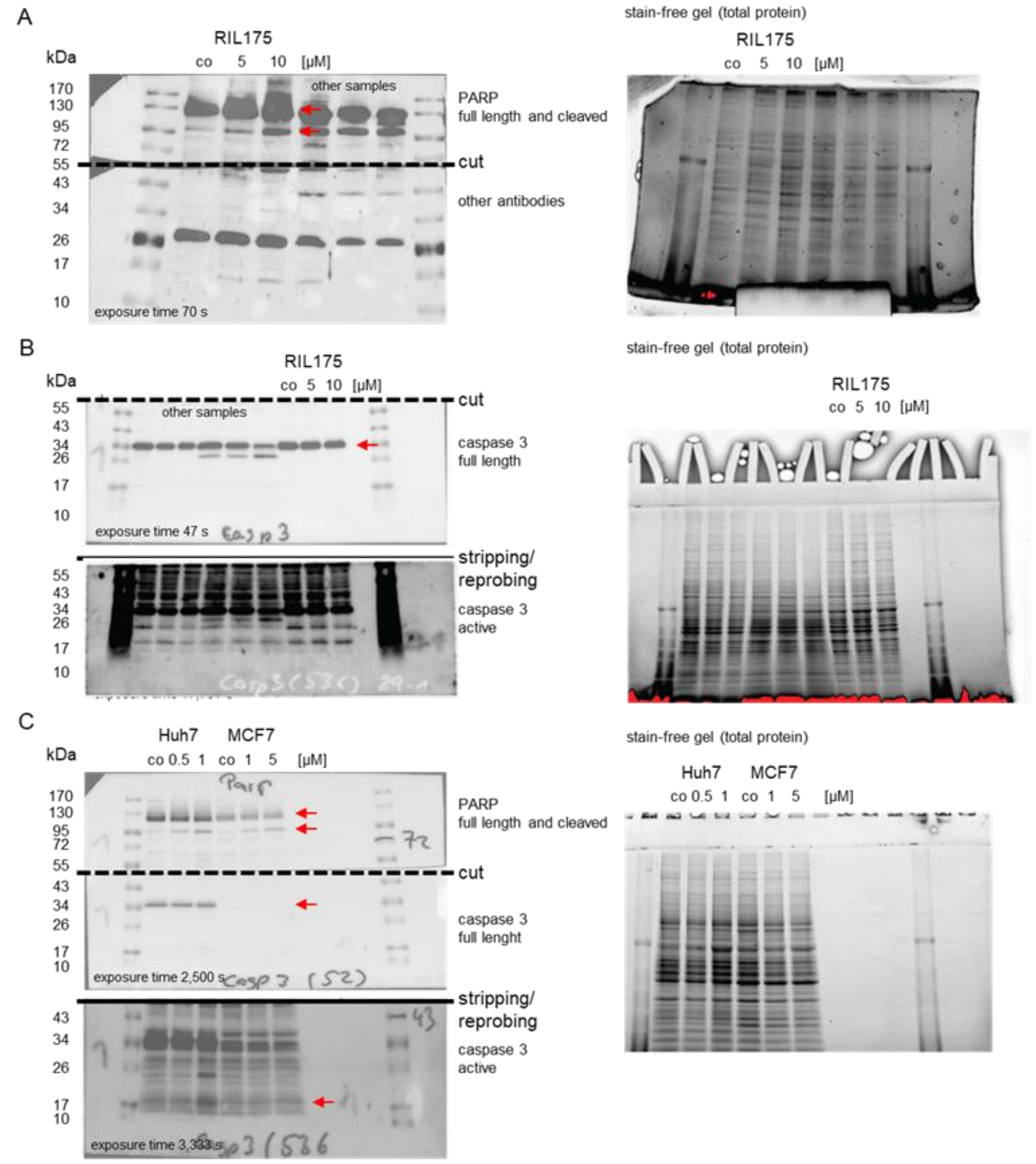

CASPASE 3 (N=1)		RELATIVE FRONT	ADJ. VOLUME	VOLUME	BAND %	LANE %	NORM. FACTOR	NORM. VOL	CASPASE 3 RATIO
RIL175	co	0,45	4711255	5208394	100,00	94,71	1,00	4711255	1,00
	5 µM	0,44	4599033	5089753	100,00	93,46	0,92	4239039	0,90
	10 µM	0,43	4294807	4776856	100,00	93,80	1,25	5389218	1,14
HUH7	co	0,24	4657716	6162760	100,00	83,92	1,00	4657716	1,00
	0.5 µM	0,24	3908774	5044790	100,00	78,35	1,18	4597367	0,99
	1 µM	0,24	3643362	4695551	100,00	74,89	0,94	3431300	0,74
**CASPASE 3 (N=2)**		**RELATIVE FRONT**	**ADJ. VOLUME**	**VOLUME**	**BAND %**	**LANE %**	**NORM. FACTOR**	**NORM. VOL**	**CASPASE 3 RATIO**
RIL175	co	0,239837	3099643	3487762	97,992	91,91540	1	3099643	1,00
	5 µM	0,243902	5198169	5661759	89,4201	79,70721	0,508792	2644786	0,85
	10 µM	0,235772	4311899	4715222	95,3132	86,68987	0,792049	3415237	1,10
HUH7	co	0,19917	2828407	3807576	95,4158	88,68724	1	2828407	1,00
	0.5 µM	0,186722	1873398	2601233	91,7473	84,05391	1,356397	2541072	0,90
	1 µM	0,20332	1717237	2362907	89,6842	79,93951	1,314732	2257706	0,80
**CASPASE 3 (N=3)**		**RELATIVE FRONT**	**ADJ. VOLUME**	**VOLUME**	**BAND %**	**LANE %**	**NORM. FACTOR**	**NORM. VOL**	**CASPASE 3 RATIO**
RIL175	co	0,377049	4404303	5053164	100	91,04595	1	4404303	1,00
	5 µM	0.239631	2571421	2764876	98.222	86.35433	0.844491	2171541	1.00
	10 µM	0.258065	2760608	3018329	97.992	91.81400	0.686159	1894212	0.87
HUH7	co	0,195122	7105988	8930624	96,6914	77,77800	0,969753	6891054	1,00
	0.5 µM	0,170732	6896364	8954081	93,9588	74,75209	0,790859	5454051	0,79
	1 µM	0,178862	6241310	8123012	93,4701	68,09081	0,766766	4785625	0,69

CASPASE 3 CLEAVED (N=1)		RELATIVE FRONT	ADJ. VOLUME	VOLUME	BAND %	LANE %	NORM. FACTOR	NORM. VOL	CLEAVED RATIO	CLEAVED/ TOTAL	CLEAVED/ TOTAL RATIO
RIL175	co	0.77	2181865	6382990	100.00	4.09	1.00	2181865	1.00	0.05	1.00
	5 µM	0.79	3900480	13180780	100.00	8.15	0.87	3408032	1.56	0.08	1.74
	10 µM	0.83	3643911	11925116	100.00	8.96	1.18	4297374	1.97	0.08	1.72
HUH7	co	0,75	5603185	17872595	100,00	5,34	1,00	5603185	1,00	0,12	1,00
	0.5 µM	0,75	6143517	17949870	100,00	6,51	1,11	6810045	1,22	0,15	1,23
	1 µM	0,75	12306504	29542504	100,00	12,15	0,82	10121644	1,81	0,29	2,45
**CASPASE 3 CLEAVED (N=2)**		**RELATIVE FRONT**	**ADJ. VOLUME**	**VOLUME**	**BAND %**	**LANE %**	**NORM. FACTOR**	**NORM. VOL**	**CLEAVED RATIO**	**CLEAVED/ TOTAL**	**CLEAVED/ TOTAL RATIO**
RIL175	co	0,857965	8210858	30470628	100	15,16024	1	8210858	1,00	0,26	1,00
	5 µM	0,846591	30526151	56118455	100	23,44805	0,506904	15473824	1,88	0,59	2,21
	10 µM	0,832386	15959636	34898094	100	20,45487	0,831978	13278059	1,62	0,39	1,47
HUH7	co	0,742739	1358906	7133173	4,584242	4,260971	1	1358906	1,00	0,05	1,00
	0.5 µM	0,717842	1685128	6362668	8,252698	7,560676	1,356397	2285702	1,68	0,09	1,87
	1 µM	0,738589	1975234	6437286	10,31583	9,194957	1,314732	2596902	1,91	0,12	2,39
**CASPASE 3 CLEAVED (N=3)**		**RELATIVE FRONT**	**ADJ. VOLUME**	**VOLUME**	**BAND %**	**LANE %**	**NORM. FACTOR**	**NORM. VOL**	**CLEAVED RATIO**	**CLEAVED/ TOTAL**	**CLEAVED/ TOTAL RATIO**
RIL175	co	0.682028	155554	1663206	0.611393	0.589852	1	155554	1.00	0.01	1.00
	5 µM	0.723502	465350	1633982	1.623516	1.562754	0.844491	392983	2.53	0.02	2.53
	10 µM	0.732719	565636	1284088	1.93988	1.88123	0.686159	388116	2.50	0.02	2.86
HUH7	co	0,78125	2801582	16368762	100	10,2093	1	2801582	1,00	0,41	1,00
	0.5 µM	0,772727	6479902	40612102	100	11,84251	0,506904	3284687	1,17	0,60	1,48
	1 µM	0,775568	4739350	33231044	100	10,0944	0,831978	3943083	1,41	0,82	2,03

PARP (N=1)		RELATIVE FRONT	ADJ. VOLUME	VOLUME	BAND %	LANE %	NORM. FACTOR	NORM. VOL	PARP RATIO
RIL175	co	0,4279	15585520	17666080	94,74	69,23	1,0000	15585520	1,00
	5 µM	0,4423	23675440	26878240	93,82	73,85	0,8445	19993696	1,28
	10 µM	0,4423	25090480	30559600	87,81	70,98	0,6862	17216059	1,10
HUH7	co	0,4288	80379096	96152616	93,15	65,79	1,0000	80379096	1,00
	0.5 µM	0,4271	76165845	89587386	85,43	59,12	1,1812	89965420	1,12
	1 µM	0,4271	66569692	78055705	77,84	49,48	0,9490	63175503	0,79
MCF7	co	0,4144	35689970	46098216	90,72	48,36	1,0642	37981266	1,00
	1 µM	0,4003	26276832	35212032	77,57	40,66	1,1911	31299149	0,82
	5 µM	0,3877	29914056	41961120	70,3	49,11	1,1754	35160084	0,93
PARP (N=2)		RELATIVE FRONT	ADJ. VOLUME	VOLUME	BAND %	LANE %	NORM. FACTOR	NORM. VOL	PARP RATIO
RIL175	co	0,447619	9815634	12594593	92,787384	69,301507	1	9815634	1,00
	5 µM	0,461538	8661425	11016408	92,247697	58,226999	0,968523	8388785	0,85
	10 µM	0,470874	3217809	5286032	86,921977	46,80538	0,960999	3092310	0,32
HUH7	co	0,339161	62705676	75848224	78,628734	65,352834	1	62705676	1,00
	0.5 µM	0,370629	37279440	48573360	63,332165	48,534112	1,356397	50565718	0,81
	1 µM	0,395105	30492000	40670280	55,574267	40,218996	1,314732	40088802	0,64
MCF7	co	0,423077	39829867	50364644	68,834048	60,82618	0,698472	27820051	1,00
	1 µM	0,440559	26404147	36151012	39,881018	36,698015	1,122259	29632287	1,07
	5 µM	0,447552	11711538	18238059	21,103272	19,417619	1,148628	13452196	0,48
PARP (N=3)		RELATIVE FRONT	ADJ. VOLUME	VOLUME	BAND %	LANE %	NORM. FACTOR	NORM. VOL	PARP RATIO
RIL175	co	0,38764	30254140	35113960	91,585296	70,881953	0,923509	27939961	1,00
	5 µM	0,365169	27524070	33002130	91,786399	61,320285	0,85453	23520146	0,84
	10 µM	0,353933	24431190	29107050	88,605934	66,752031	1,164869	28459128	1,02
HUH7	co	0,389706	63522556	65905071	91,721226	68,65368	1	63522556	1,00
	0.5 µM	0,400735	11041158	12288565	72,088701	45,61199	2,735623	30204451	0,48
	1 µM	0,404412	22089288	27418967	71,126267	55,020881	1,224848	27056030	0,43
MCF7	co	0,404412	30811758	34725042	83,512405	65,606062	0,90978	28031906	1,00
	1 µM	0,397059	24524425	28375708	61,23555	51,384901	1,078117	26440194	0,94
	5 µM	0,375	19343316	24152668	50,87694	46,502793	1,121322	21690087	0,77

PARP CLEAVED (N=1)		RELATIVE FRONT	ADJ. VOLUME	VOLUME	BAND %	LANE %	NORM. FACTOR	NORM. VOL	PARP CLEVED RATIO	CLEAVED /TOTAL PARP	RATIO CLEAVED PARP/TO
RIL175	co	0,6538	864240	1886080	5,25	3,84	1,0000	864240	1,00	0,055	1,00
	5 µM	0,6587	1559840	3126960	6,18	4,87	0,8445	1317271	1,52	0,066	1,19
	10 µM	0,6490	3480400	6146320	12,18	9,85	0,6862	2388108	2,76	0,139	2,50
HUH7	co	0,5579	5909904	14115696	6,85	4,83	1,0000	5909904	1,00	0,074	1,00
	0.5 µM	0,5611	12988773	21454062	14,57	10,08	1,1812	15342053	2,60	0,171	2,32
	1 µM	0,5627	18951156	25511528	22,16	14,09	0,9490	17985294	3,04	0,285	3,87
MCF7	co	0,5753	3650947	12137093	9,28	4,95	1,0642	3885338	1,00	0,102	1,00
	1 µM	0,5511	7597640	13386208	22,43	11,76	1,1911	9049785	2,33	0,289	2,83
	5 µM	0,5368	12639104	20607552	29,71	20,75	1,1754	14855624	3,82	0,423	4,13
PARP CLEAVED (N=2)		RELATIVE FRONT	ADJ. VOLUME	VOLUME	BAND %	LANE %	NORM. FACTOR	NORM. VOL	PARP CLEVED RATIO	CLEAVED /TOTAL PARP	RATIO CLEAVED PARP/TO
RIL175	co	0,690476	762996	2515783	7,212616	5,386995	1	762996	1,00	0,078	1,00
	5 µM	0,706731	727888	2308485	7,752303	4,893275	0,968523	704975	0,92	0,084	1,08
	10 µM	0,703883	484142	1903001	13,07802	7,042199	0,960999	465259	0,61	0,150	1,94
HUH7	co	0,552448	17043384	26265856	21,37126	17,76288	1	17043384	1,00	0,272	1,00
	0.5 µM	0,58042	21583920	29361000	36,66783	28,10011	1,356397	29276363	1,72	0,579	2,13
	1 µM	0,604895	24375120	31968000	44,42573	32,15082	1,314732	32046745	1,88	0,799	2,94
MCF7	co	0,636364	18033746	25741249	31,16595	27,54023	0,698472	12596068	1,00	0,453	1,00
	1 µM	0,65035	39803158	49808816	60,11898	55,32073	1,122259	44669445	3,55	1,507	3,33
	5 µM	0,657343	43784775	52801524	78,89672	72,59474	1,148628	50292404	3,99	3,739	8,26
PARP CLEAVED (N=3)		RELATIVE FRONT	ADJ. VOLUME	VOLUME	BAND %	LANE %	NORM. FACTOR	NORM. VOL	PARP CLEVED RATIO	CLEAVED /TOTAL PARP	RATIO CLEAVED PARP/TO
RIL175	co	0,696629	2779700	4637080	8,414704	6,512516	0,923509	2567077	1,00	0,092	1,00
	5 µM	0,691011	2463020	5633670	8,213601	5,487309	0,85453	2104724	0,82	0,089	0,97
	10 µM	0,674157	3141670	5986890	11,39406	8,583817	1,164869	3659633	1,43	0,129	1,40
HUH7	co	0,547794	5733557	6692871	8,278774	6,196693	1	5733557	1,00	0,090	1,00
	0.5 µM	0,558824	4274915	5669647	27,91129	17,66004	2,735623	11694557	2,04	0,387	4,29
	1 µM	0,5625	8967154	12906749	28,87373	22,33574	1,224848	10983404	1,92	0,406	4,50
MCF7	co	0,566176	6083070	9572556	16,48759	12,95240	0,90978	5534252	1,00	0,197	1,93
	1 µM	0,555147	15524901	20330435	38,76445	32,52861	1,078117	16737656	3,02	0,633	6,19
	5 µM	0,533088	18676494	23313538	49,12306	44,89970	1,121322	20942365	3,78	0,966	9,44

Figure S4. Whole blots and corresponding stain-free gels with densitometric readings summarized in Figure 2 D–F. RIL175 cell lysate, stained for PARP and cleaved PARP (**A**). RIL175 cell lysate, stained for caspase 3 (upper panel) and cleaved caspase 3 (lower panel; **B**). Huh7 and MCF7 cell lysate stained for PARP and cleaved PARP (upper panel), caspase 3 (middle panel) and cleaved caspase 3 (lower panel; **C**). Representative pictures are shown. Red arrows indicate the respective bands. All densitometric values and factors were calculated by Image Lab™ software and are demonstrated in tables (Relative front: indicates the relative movement of the band from top to bottom, adj. volume: the volume with background subtracted, volume: the sum of all the intensities in the volume, band %: percentage of the band´s volume compared to all band volumes in the lanes, lane %: percentage of the band's volume compared to the entire volume of the lane, norm. factor: the correction factor for the lane that contains the band, quantified by total protein loading, norm. vol. (Int): the adjusted volume corrected by the normalization factor, Ratio: Normalization to control samples).

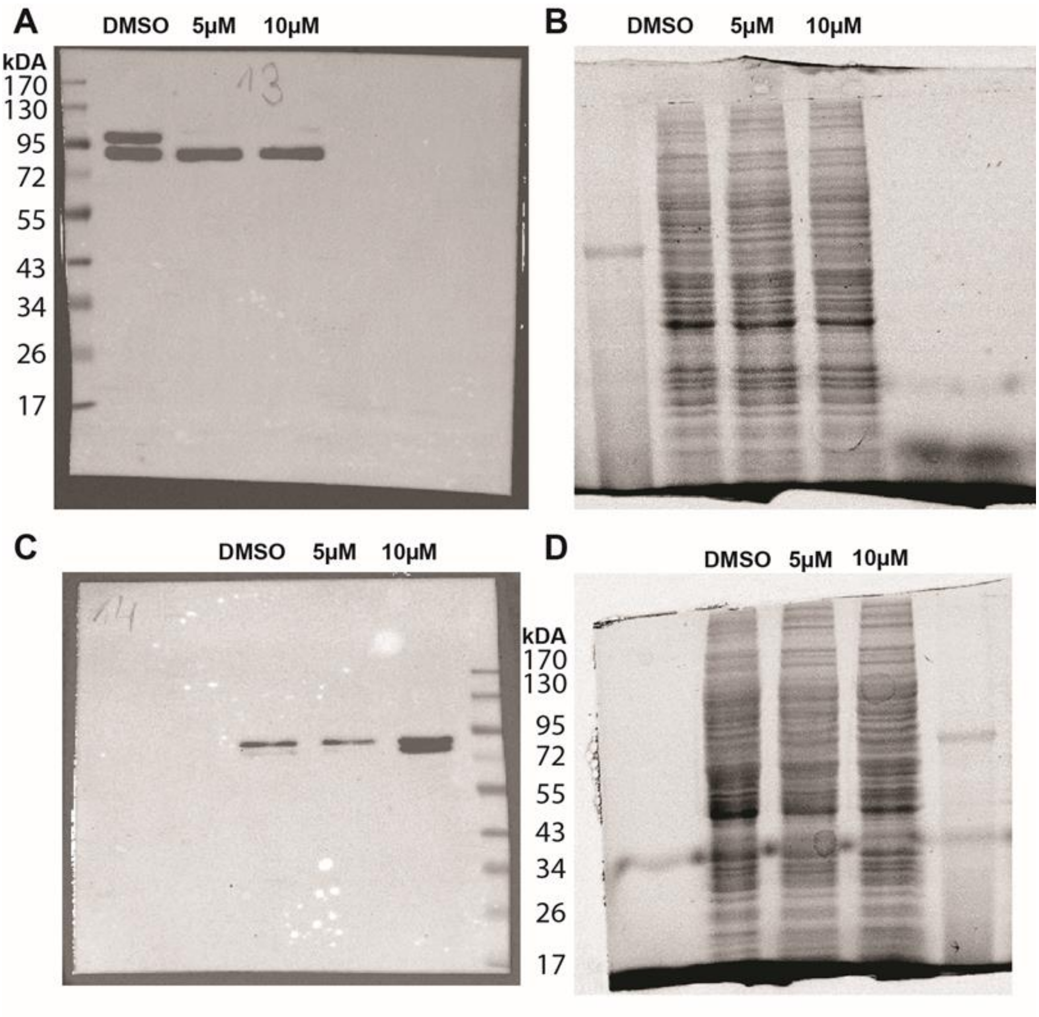

L-OPA-1		relative front	adj. Volume	volume	band %	lane %	norm. factor	norm. vol (int)	L-OPA ratio
n1	co	0,326531	26985312	36166248	41,70587	39,55143	1,108347	29909079	1,00
	5 µM	0,300454	388725	8264841	1,246147	1,137106	1	388725	0,01
	10 µM	0,291383	324722	6500394	1,263015	1,143622	1,062621	345056	0,01
n2	co	0,37486	19891989	28560051	34,86046	31,8949	1	19891989	1,00
	5 µM	0,379349	80080	5743374	2,714039	1,403374	1,917338	153540	0,01
	10 µM	0,397489	57855	4497668	5,28757	1,681019	1,147865	66409	0,00
n3	co	0,331995	35418990	45630250	37,34843	35,44453	0,884642	31333110	1,00
	5 µM	0,356091	23750	1155390	6,062076	0,626692	1	23750	0,00
	10 µM	0,370817	16340	1040084	0,181811	0,141466	0,730678	11939	0,00

S_OPA-1		relative front	adj. Volume	volume	band %	lane %	norm. Factor	norm. Vol (int)	SOPA ratio	S/L	S/L ratio
n1	co	0,388889	37718560	48307968	58,29413	55,28277	1,108347	41805238	1,00	1,397744	1
	5 µM	0,393424	30805416	40697208	98,75385	90,11262	1	30805416	0,74	79,24732	56,69659
	10 µM	0,390023	25385337	35155393	98,73699	89,40335	1,062621	26974994	0,65	78,1757	55,92991
n2	co	0,426487	37169769	46602049	65,13954	59,59816	1	37169769	1,00	1,86858	1
	5 µM	0,435466	2870504	9486022	97,28596	50,3046	1,917338	5503726	0,15	35,84555	19,18331
	10 µM	0,434343	1036315	5068707	94,71243	30,11089	1,147865	1189549	0,03	17,91247	9,586139
n3	co	0,427042	59414970	71355240	62,65158	59,45782	0,884642	52560952	1,00	1,677489	1
	5 µM	0,448461	368030	1513920	93,93792	9,71122	1	368030	0,01	15,496	9,237616
	10 µM	0,453815	8971004	19066888	99,81819	77,66775	0,730678	6554916	0,12	549,0339	327,2951

DRP1		relative front	adj. Volume	volume	band %	lane %	norm. Factor	norm. Vol	Drp-1 ratio
n1	co	0,400181	4646400	11478480	86,78501	48,00397	1	4646400	1,00
	5 µM	0,391147	3054708	8283052	97,69476	42,67816	1,920989	5868060	1,26
	10 µM	0,390244	11643400	19038200	62,19831	52,46098	1,08087	12584996	2,71
n2	co	0,28357	9305776	16825700	66,39684	43,24983	1	9305776	1,00
	5 µM	0,276897	1511714	7979834	98,7452	23,21199	2,112351	3193270	0,34
	10 µM	0,277731	21824368	32780170	81,36359	65,57609	1,152887	25161029	2,70

Figure S5. Whole blots and corresponding stain-free gels with densitometric readings summarized in Figure 3 G–K. RIL175 cell lysate, stained for L-OPA1 (**A**, upper band) and S-OPA1 (A, lower band) and corresponding stain-free gel (**B**). RIL175 cell lysate, stained for DRP1 (**C**) and corresponding stain-

free gel (**D**). Representative pictures are shown. All densitometric values and factors were calculated by Image Lab™ software and are demonstrated in tables (Relative front: indicates the relative movement of the band from top to bottom, adj. volume: the volume with background subtracted, volume: the sum of all the intensities in the volume, band %: percentage of the band´s volume compared to all band volumes in the lanes, lane %: percentage of the band's volume compared to the entire volume of the lane, norm. factor: the correction factor for the lane that contains the band, quantified by total protein loading, norm. vol. (Int): the adjusted volume corrected by the normalization factor, Ratio: Normalization to control samples).

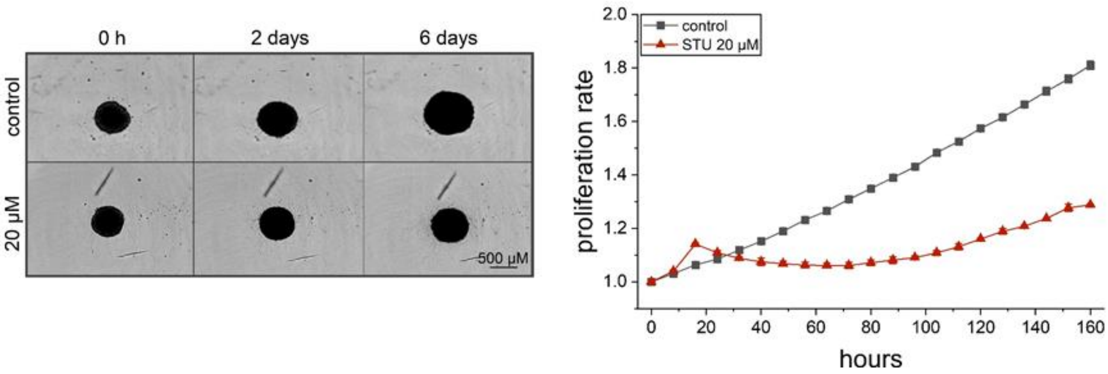

Figure S6. Staurosporine-induced effects on proliferation in 3D cell culture. 3-day old HCT116 tumor spheroids were treated with 20 µM staurosporine or vehicle control, and the spheroid area was analyzed by automated microscopy (right panel; *n* = 2(4)). Spheroids are shown in representative pictures at the starting point and the time points 2 days and 6 days after treatment (left panel). *n* = 3 (quadruplicates).

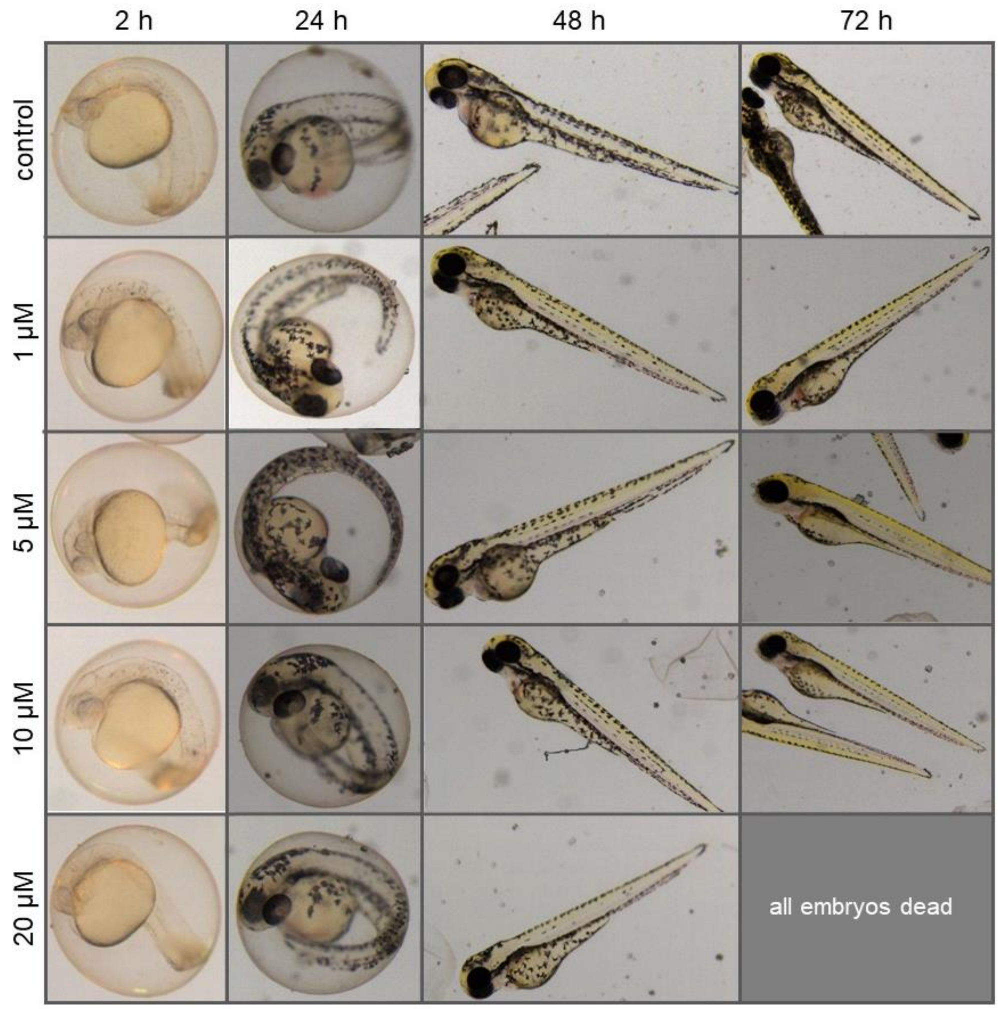

Figure S7. ThioA effects on zebrafish embryo development and viability. Embryos at 24 hpf were treated with thioA or vehicle control in the fish water. Eye, heart, and body axis formation, heartbeat, and pigmentation were observed during 72 h treatment. Representative pictures are shown.

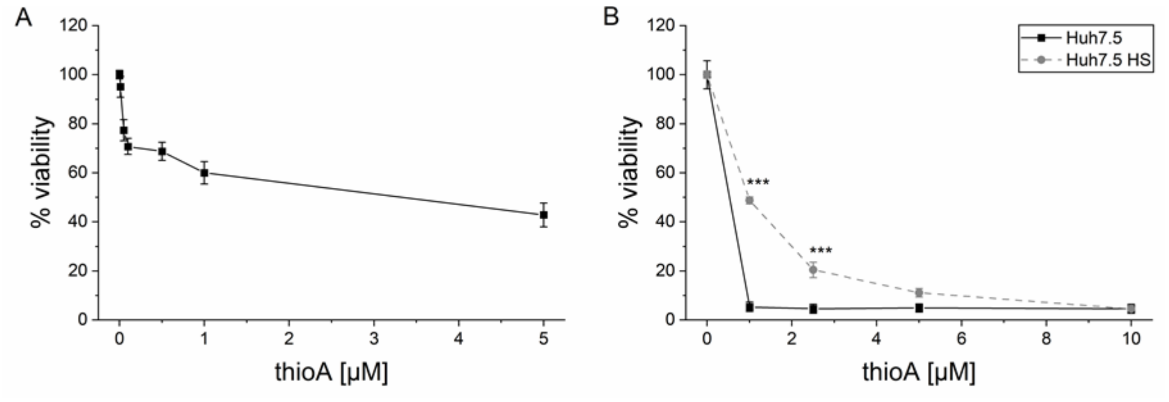

Figure S8. ThioA-induced effects on normal cell viability. HUVEC (**A**) and human serum-differentiated Huh7.5 (**B**) cells were treated with increasing concentrations of the compound, and cell viability was determined after 48 h treatment in an MTT assay. Vehicle control-treated cells were used for data normalization. Statistical analysis was performed for the respective concentrations using one-way ANOVA followed by Bonferroni's post-hoc analysis. $n = 3$ (quadruplicates).

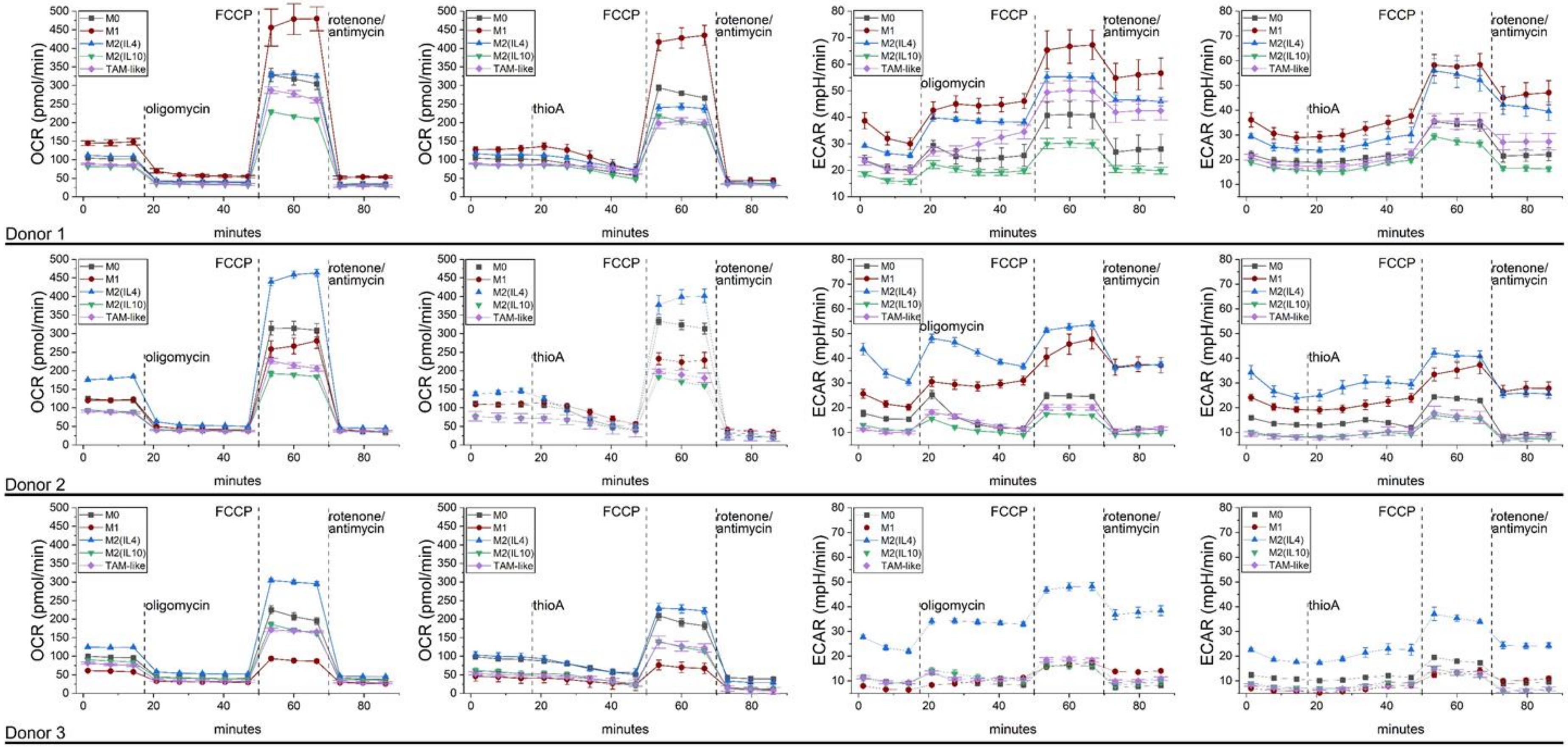

Figure S9. ThioA affects the metabolism of in vitro differentiated and polarized macrophages; donor specific differences. HMDMs were polarized into M0, M1, M2(IL4), M2(IL10), and TAM-like macrophages for 24 h. OCR and ECAR were measured in a mito stress test using a Seahorse 96XF instrument. 1 µM oligomycin was injected to shut down OXPHOS-dependent ATP production or 1 µM thioA was injected instead. Values are shown for three individual donors. n = 3 (quadruplicates).

(A)

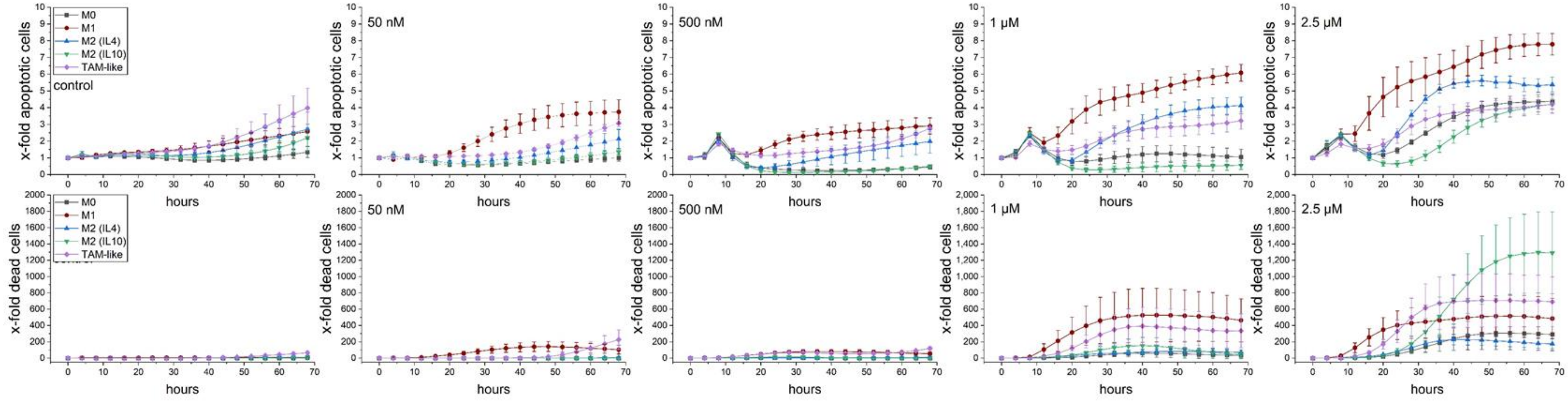

(B)

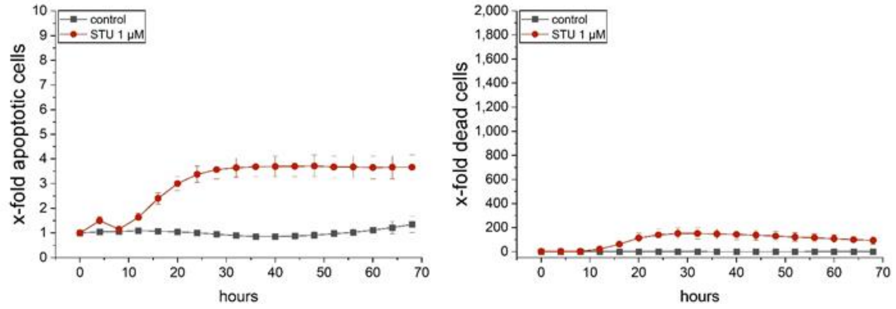

Figure S10. Assessment of toxic concentrations of thioA in HMDMs. HMDMs were polarized in vitro into M0, M1, M2(IL4), M2(IL10), and TAM-like macrophages for 24 h. Polarized macrophages were stained for caspase 3/7 activity and cell membrane permeability and analyzed in an IncuCyte system during thioA or vehicle control treatment for 68 h (**A**). M0 macrophages were treated with 1 µM staurosporine or vehicle control (**B**). Fluorescent signals from apoptotic and permeable cells were normalized to cell confluency. *n* = 3 (quadruplicates).

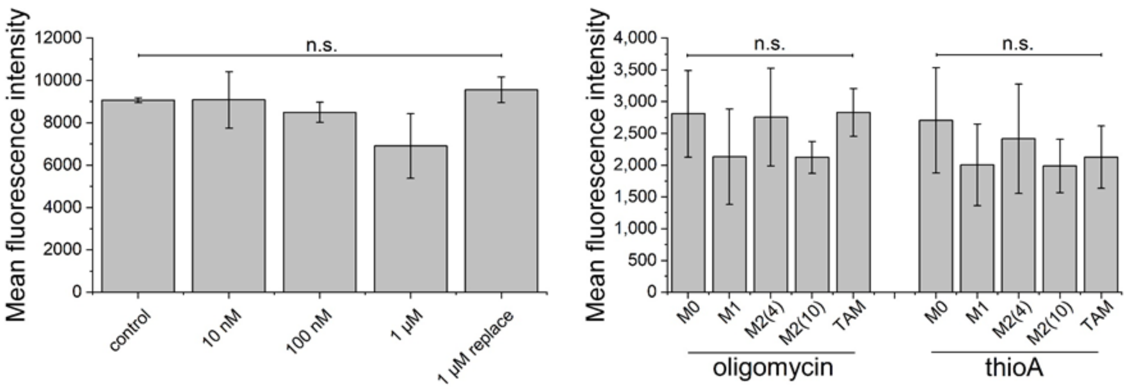

Figure S11. Cell quantification after Seahorse experiments by Hoechst staining. Tumor cells (left panel, corresponds to Figure 3) and macrophages (right panel, corresponds to Figure 7) were stained after Seahorse measurements, and mean fluorescence intensity was analyzed to ensure an equal cell distribution after different treatment steps. Statistical analysis was performed one-way ANOVA followed by Tukey´s post-hoc analysis.

© 2019 by the authors. Licensee MDPI, Basel, Switzerland. This article is an open access article distributed under the terms and conditions of the Creative Commons Attribution (CC BY) license (http://creativecommons.org/licenses/by/4.0/).
